# Venom Variation as a Window into the Ecology and Evolution of Snakes

**DOI:** 10.1093/icb/icag056

**Published:** 2026-05-26

**Authors:** Neil R Balchan, Stephen P Mackessy, Guinevere O U Wogan, Damien Esquerré, Ignazio Avella

**Affiliations:** Department of Biology, Oklahoma State University, Stillwater, Oklahoma 74078, USA; Department of Biological Sciences, University of Northern Colorado, Greeley, Colorado 80639, USA; Department of Biology, Oklahoma State University, Stillwater, Oklahoma 74078, USA; Environmental Futures Research Centre, School of Science, University of Wollongong, Wollongong, New South Wales 2500, Australia; Animal Venomics Lab, Fraunhofer Institute for Molecular Biology and Applied Ecology (IME), 35392 Giessen, Germany; Institute for Insect Biotechnology, Justus Liebig University of Giessen, Heinrich-Buff Ring 26-32, 35392 Giessen, Germany

## Abstract

Snake venoms are complex biochemical systems that function primarily in prey subjugation and defense, yet their composition varies extensively across individuals, populations, species, and environments. This variation provides a powerful framework for investigating ecological and evolutionary processes. Here, we offer a forward-looking synthesis of snake venom diversity that proposes new research directions and highlights how venom variation can illuminate eco-evolutionary dynamics across biological scales. We review evidence for 10 key contexts in which venom variation arises, including within-population differences, sexual dimorphism, geographic structuring, ontogenetic shifts, seasonal changes, interspecific divergence, hybridization, convergent evolution, prey specificity, and venom resistance. Together, these processes demonstrate that venom phenotypes are shaped by interacting selective pressures such as trophic ecology, predator–prey coevolution, environmental heterogeneity, and gene flow. While phylogenetic history establishes broad toxin composition patterns, ecological factors frequently drive rapid and repeated shifts in venom phenotype. We further outline the historical development of venom research, from early descriptive studies to modern integrative approaches enabled by advances in proteomics, transcriptomics, genomics, and functional assays. These methodological innovations increasingly allow venom composition to be linked directly to ecological performance and evolutionary outcomes. Despite this progress, major gaps remain, including limited taxonomic coverage, incomplete integration of ecological data, and insufficient experimental tests of adaptive hypotheses. Future research combining molecular, functional, and field-based approaches will be essential for resolving the mechanisms that generate and maintain venom diversity. As complex traits shaped by interacting ecological and evolutionary forces, snake venoms provide an exceptional model system for understanding how selection, constraint, and environmental context interact to produce phenotypic diversity across time and space.

## Introduction

**Fig. 1:**
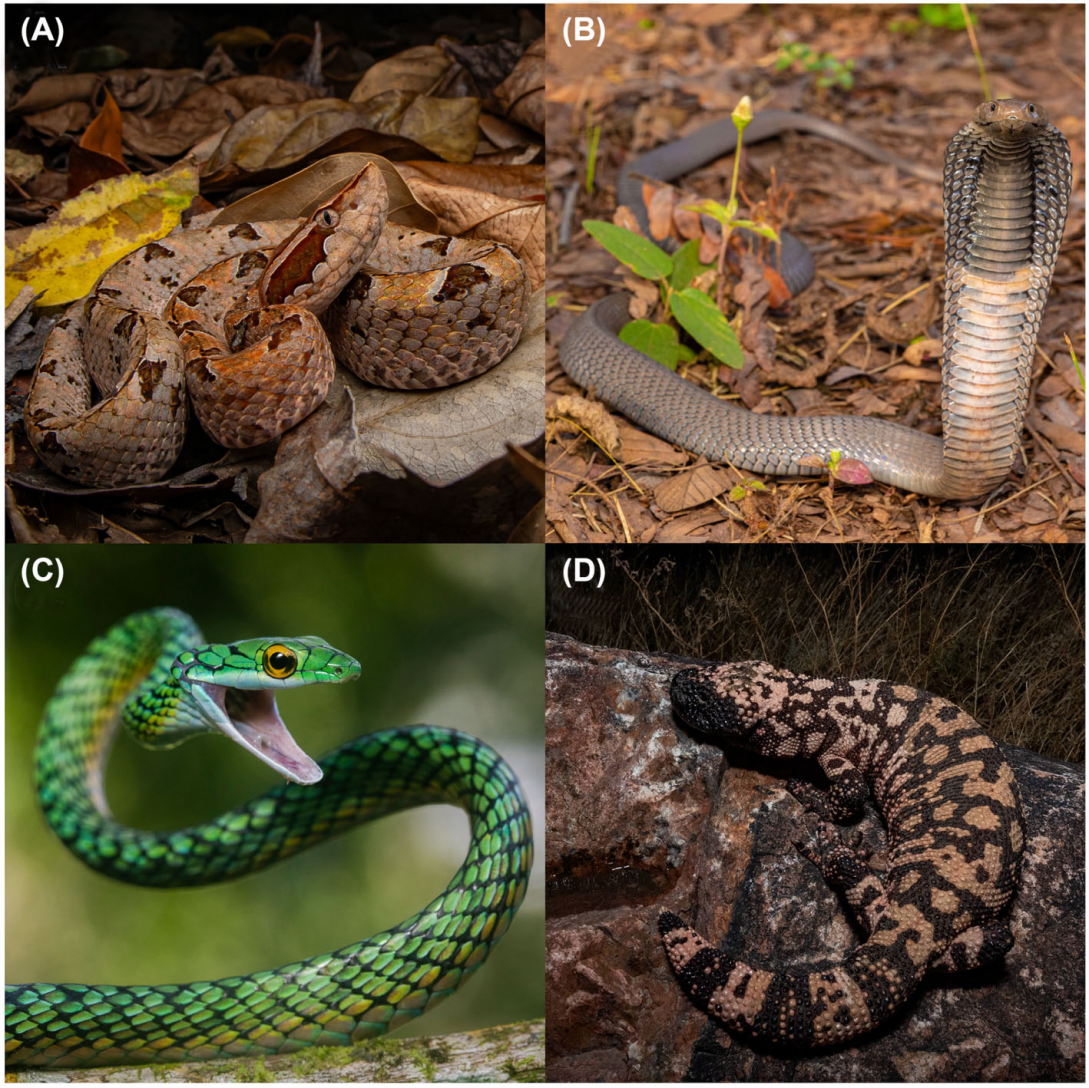
Venomous species exist across the squamate tree of life in multiple lineages. (A) *Calloselasma rhodostoma* is a pit viper distributed across Southeast Asia with venoms adapted for local prey communities, and the link between snake venom and diet was thoroughly investigated for the first time in this species (Daltry et al. 1996). (B) *Naja nigricollis* is an elapid snake from Sub-Saharan Africa with the ability to spit venom defensively (Kazandjian et al. 2021). (C) *Leptophis ahaetulla* is a venomous arboreal colubrid from South America with low toxicity toward mammals (Sánchez et al. 2018). (D) *Heloderma suspectum* is a venomous anguimorph lizard native to the warm deserts of North America, with venom used primarily for defensive purposes and serving secondary roles in prey incapacitation (Beck, 2005). Photos by Artur Tomaszek (A), Chad Keates (B), Damien Esquerré (C), and Bryan Hughes (D), and used with permission.

Venoms are complex biochemical innovations whose variation illuminates ecological and evolutionary processes across species and populations (Arbuckle 2015). In squamate reptiles (Fig. 1), venoms primarily facilitate prey capture and defence and consist of diverse mixtures of proteins, peptides, and other bioactive components produced and stored in specialised glands (Chan et al. 2016; Mackessy 2022). Venomous snakes occur across several lineages, spanning the families Elapidae, Viperidae, Atractaspididae, and Colubridae *sensu lato* (Fry et al. 2006; Quijada-Mascareñas and Wüster 2009; Zheng and Wiens 2016). Although this review focuses on snakes, venoms are also present in anguimorph lizards of the genus *Heloderma* (Fry et al. 2010; Sanggaard et al. 2015), which are discussed where relevant; their presence in *Varanus* remains debated (Fry et al. 2006; Hargreaves et al. 2014; Sweet 2016).

The extraordinary diversity of snake venom components is widely attributed to the “birth-and-death” model of gene evolution (Nei et al. 1997; Fry et al. 2003). Under this model, genes encoding physiological proteins undergo duplication, producing multiple copies that evolve under relaxed functional constraint. Although many duplicates accumulate deleterious mutations and become pseudogenes, others are retained and may become selectively expressed in the venom gland (Ohno 1970). These retained duplicates frequently undergo further rounds of duplication and divergence, producing large toxin gene families with substantial structural and functional diversity (Kordiš and Gubenšek 2000; Fry et al. 2003; Chang and Duda 2012; Casewell et al. 2013). Additional diversification can arise through mechanisms such as alternative and trans-splicing, particularly within toxin families including snake venom metalloproteinases (SVMPs), serine proteinases, and vascular endothelial growth factors (Shibata et al. 2018; Ogawa et al. 2019). Consistent with these dynamics, venom toxin genes commonly exhibit signatures of accelerated molecular evolution (Ohno et al. 2003; Ogawa et al. 2005; Doley et al. 2009) and positive selection (Gibbs and Rossiter 2008; Juarez et al. 2008; Rokyta et al. 2011), especially at surface-exposed residues that mediate protein–target interactions while preserving structural stability (Casewell et al. 2011; Sunagar and Moran 2015).

This evolutionary framework produces extensive venom variation across taxonomic scales. Differences in toxin composition and abundance reflect lineage-specific histories as well as ecological and selective pressures shaping toxin expression and deployment (Casewell et al. 2020). Diet is widely considered a major driver of venom evolution (Davies and Arbuckle 2019; Holding et al. 2021; Siqueira‐Silva et al. 2021) Species with highly specialized diets often possess relatively simple venoms; for example, many sea snakes (Elapidae) feed primarily on fish and typically express venoms dominated by three-finger α-neurotoxins and phospholipases A₂ (Mackessy and Tu 1993; Li et al. 2005; Wang et al. 2020). In contrast, many terrestrial front-fanged snakes exhibit biochemically complex venoms containing dozens of proteins and peptides from multiple toxin families (Oliveira et al. 2022). Variation in toxin composition and relative abundance can therefore substantially alter venom effects (Table 1), generating extensive phenotypic diversity within and among species.

**Table 1:** List of toxin families commonly found in squamate venoms, with major venom toxins bolded. Taxonomic clades possessing these toxins in large proportions are indicated (V = Viperidae, E = Elapidae, C = Colubridae, H = Heloderma) in addition to function of toxins and common biological effects. For a more complete review of toxins comprising squamate venoms and references, see Mackessy (2010a, 2021).

Toxin	Clades	Functions	Common biological effects	References
5′-nucleotidase (5N)	E, V	Nucleotide breakdown	Hypotension, reduced platelet aggregation, circulatory collapse contributing to immobilization	Dhananjaya and D’Souza (2010), Mackessy (2021)
Acetylcholinesterase (Ache)	C, E	Hydrolysis of acetylcholine	Depletion of neurotransmitters; tetanic paralysis	Mackessy (2010a, 2021), Frobert et al. (1997)
Aminopeptidase (AmPep)	E, V	Protein degradation	Degradation of regulatory peptides, tissue damage, interference with physiological signaling	Mackessy (2010a, 2021), Vaiyapuri et al. (2010)
Bradykinin-potentiating peptides (BPP)	V	Increases potency of bradykinin	Pain, hypotension, prey immobilization	Mackessy (2010a, 2021), Sciani and Pimenta (2017)
C-type lectins and C-type lectin-related proteins (CTL)	V	Bind to platelet and collagen receptor	Haemostasis disruption, platelet modulator	Mackessy (2010a, 2021), Morita (2005), Ogawa et al. (2005), Arlinghaus and Eble (2012)
Cobra venom factor (CVF)	E	Complement system activator that forms stable C3/C5 convertase	Complement depletion, immune disruption, increased inflammation	Kock et al. (2004), Vogel and Fritzinger (2010)
Cystatin (Cys)	E, V	Protease inhibition	Modulates proteolysis in venom; may protect other toxins and contribute to tissue disruption	Mackessy (2010a, 2021), Richards et al. (2011)
Cystein-rich secretory proteins (CRISP)	H, C, E, V	May induce hypothermia	Lethargy, paralysis, prey capture/immobilization	Mackessy (2010a, 2021), Tadokoro et al. (2020)
Disintegrins (Dis)	V	Inhibit binding of integrins to receptors	Anti-angiogenic and haemostasis-altering effects	Mackessy (2010a), Calvete et al. (2005, 2021), Calvete (2013), Almeida et al. (2023)
Hyaluronidase (Hyal)	H, E, V	Hydrolysis of interstitial hyaluronan	Decreased interstitial viscosity	Girish et al. (2002), Mackessy (2010a, 2021)
Kunitz-type protease inhibitor (Kun)	E, V	Protease inhibition, ion channel blocking	Neurotoxicity, paralysis, disruption of nerve signaling	Mackessy (2010a, 2021), Mukherjee et al. (2014)
L-amino-acid oxidase (LAAO)	E, V	Oxidative deamination of L-amino acids	Induces apoptosis, cell damage	Du and Clemetson (2002), Mackessy (2010a, 2021), Guo et al. (2012), Izidoro et al. (2014)
Natriuretic peptide (NP)	E, V	Cardiovascular regulation and vasodilation	Vasodilation, hypotension, disruption of blood pressure, rapid prey weakening	Schweitz et al. (1992), Mackessy (2010a, 2021), Vink et al. (2012)Ang et al. (2012)
Nerve growth factor (NGF)	E, V	Affects nerve cells and mast cell activation	Pain induction, inflammation, vascular permeability changes	Kostiza and Meier (1996), Mackessy (2010a, 2021), Trummal et al. (2011)
Ohanin/Vespryn (Oha-Vesp)	E	Disruption of nervous system and immobilization	Hypolocomotion, disorientation, reduced motor control	Pung, et al. (2005)
Peptide myotoxins (crotamine, myotoxin a, etc.)	V	Ion channel inhibitors	Rapid immobilization via tetanic hyperextension	Oguiura et al. (2005), Mackessy (2010a, 2021), Kerkis et al. (2014)
Phosphodiesterase (PDE)	C, E, V	Degrades phosphodiester bonds in nucleotides and nucleic acids	Hypotension, shock, disruption of cellular signaling, contributes to systemic toxicity	Dhananjaya and D’souza (2010), Mackessy (2010a, 2021)
**Phospholipase A₂ (PLA₂)**	H, C, E, V	Ca^2+^-dependent hydrolysis of 2-acyl groups in 3-sn-phosphoglycerides	Myotoxicity, myonecrosis, lipid membrane damage	Kini (2003), Mackessy (2010a, 2021)
**PLA₂-based presynaptic neurotoxins**	E, V	Blocks release of acetylcholine from axon terminus	Potent neurotoxicity, prey immobilization	Montecucco et al. (2008), Mackessy (2010a, 2021)
Phospholipase B (PLB)	E, V	Hydrolyzes phospholipids at multiple positions in membranes	Membrane disruption, hemolysis, tissue damage	Bernheimer, et al (1987), Ullah, 2020 and Masood (2020)
Phospholipase C (PLC)	E	Cleaves phospholipids to produce diacylglycerol and phosphorylated head groups	Cell membrane breakdown, inflammation, tissue necrosis	Huang, et al. (1995)
**Serine proteases (SVSP)**	H, C, E, V	Kallikrein-like; releases bradykinin, Thrombin like; catalysis of fibrinogen, and various others	Rapid hypotension, coagulopathy	Matsui et al. (2000), Kini (2005), Mackessy (2010a, 2021)
**Snake venom metalloproteinases (SVMP)**	C, V	Hydrolysis of structural proteins including basal lamina	Haemorrhage, necrosis and tissue destruction, possible prey predigestion	Gutiérrez (2000)Teixeira et al. (2005), Fox and Serrano (2008), Mackessy (2010a, 2021)
**Three-finger toxins (3FTx)**	C, E	Potent inhibitor of neuromuscular transmission, cytotoxicity, enzyme inhibiting effects, often taxon-specific effects	Flaccid paralysis, rapid prey immobilization/death	Mackessy (2010a, 2021), Sunagar et al. (2013), Utkin (2019)
Whey acidic protein motif protease inhibitor (Waprin)	E, V	Small secreted protease inhibitor with antimicrobial activity	May disrupt microbial defense or modulate proteolysis, minor direct toxicity	Nair, et al. (2007)

Given this diversity, snake venoms have long attracted scientific interest. Historically, much venom research has been motivated by clinical and human health considerations (e.g., Sintiprungrat et al. 2016). More recently, studies have increasingly examined venom variation through ecological and evolutionary perspectives (Barlow et al. 2009; Jackson et al. 2016; Sunagar et al. 2016; Zancolli et al. 2016; Strickland et al. 2018; Barua and Mikheyev 2019; Tasoulis et al. 2020; Smith et al. 2023a) Despite this progress, integrative eco-evolutionary analyses remain comparatively limited. Here, we provide a forward-looking perspective that synthesizes the current state of the field, highlighting ten ecological and evolutionary processes illuminated by snake venom variation. We emphasize that this framework is not exhaustive but rather intended as a conceptual springboard for future research. We also summarize methodological approaches used in venom research and outline promising directions for advancing this field.

## Venoms as complex traits in the eco-evolutionary arena

**Fig. 2:**
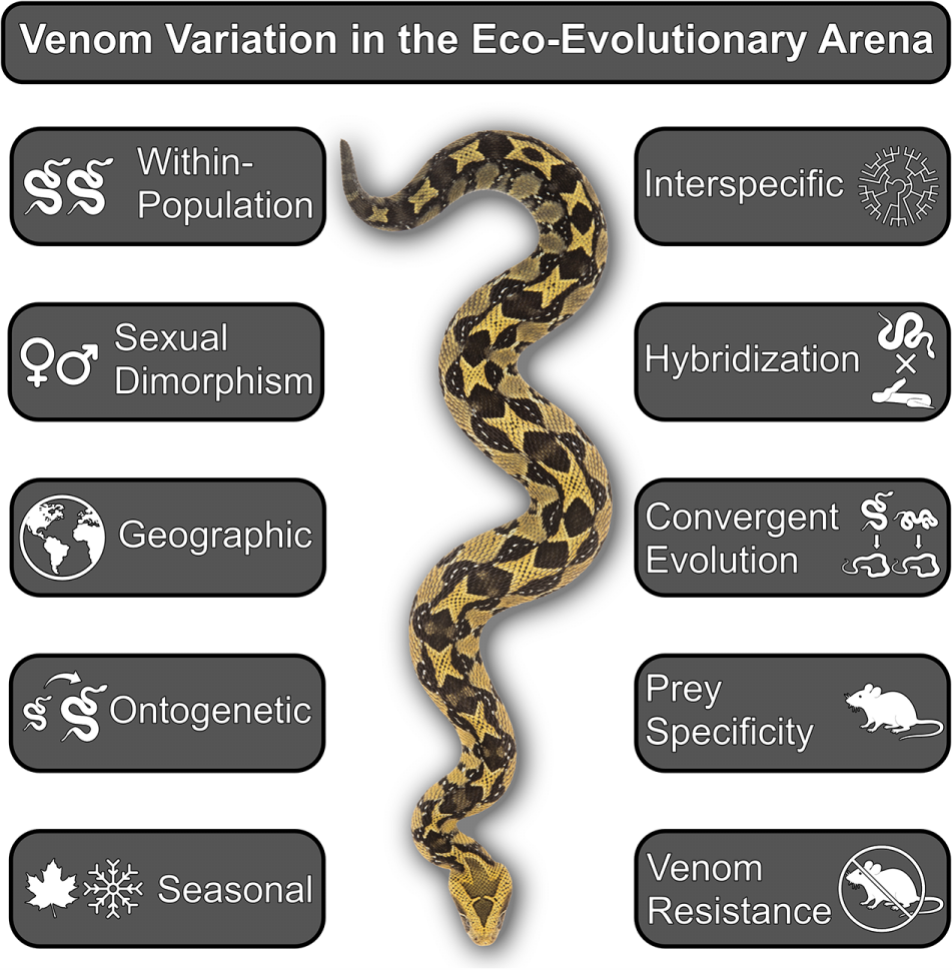
Snake venoms may show compositional variation in response to a variety of ecological and evolutionary processes. Indicated here are different processes that have been studied, and hold promise for future investigation. Photo of *Bitis parviocula* by Thor Håkonsen and used with permission.

Diet is a primary driver of snake venom evolution and variation (Daltry et al. 1996; Barlow et al. 2009; Holding et al. 2021). Because venom functions mainly in prey subjugation, its composition closely reflects a species’ trophic ecology and is expected to experience strong natural selection. Geographic variation may track differences in local prey communities (Holding et al. 2018), ontogenetic shifts often parallel age-related dietary changes (e.g., Avella et al. 2022a), and seasonal variation can reflect temporal prey fluctuations (Christian et al. 2007) Supporting this, multiple studies document increased prey-specific toxicity, enhancing foraging efficiency (Barlow et al. 2009; Gibbs and Mackessy 2009). Lineages that shifted to prey not requiring envenomation, such as eggs or molluscs, often reduce or lose venom components as seen in *Aipysurus eydouxii* (Mackessy and Tu 1993) and *Dasypeltis scabra* (Fry et al. 2008). While prey capture remains the main selective force shaping venom composition, it also functions defensively, as exemplified by venom-spitting in African and Asian elapid snakes (*Naja* spp. and *Hemachatus* spp.; Kazandjian et al. 2021). Furthermore, the evolution of venom resistance in predators of venomous snakes (Van Thiel et al. 2022), innate avoidance of characteristic warning color patterns (Smith 1977), and emergence of Batesian mimicry in some nonvenomous taxa (Davis Rabosky et al. 2016) collectively support a secondary defensive function of venom. These patterns show that venom evolution is shaped by trophic specialization, ecological context, and multifunctional selective pressures. Below, we outline 10 eco-evolutionary contexts linking venom variation to ecological and evolutionary processes in squamates (Fig. 2).

### Within-population variation

Increasing attention has focused on variation among individuals within single populations. In many rattlesnake species, venom composition appears relatively consistent among individuals (Mackessy et al. 2003; Smith et al. 2023a; Balchan et al. 2025b), although this pattern is not universal. In regions where divergent venom phenotypes meet, populations may harbor substantial individual diversity (Strickland et al. 2018; Smith et al. 2023a). High individual variation has also been documented in the absence of geographic transitions. For example, pygmy rattlesnakes (*Sistrurus miliarius*) from a Florida population show pronounced variation in venom toxicity toward lizard prey, suggesting adaptive significance at fine ecological scales (Smiley-Walters et al. 2019). Similarly, monocled cobras (*Naja kaouthia*) from West Bengal, India, exhibit striking interindividual differences in venom composition with likely functional consequences (Rashmi et al. 2021).

Within-population venom variation remains poorly studied in colubrid snakes. However, data from colubrine (Mackessy et al. 2006), dipsadine (Tioyama et al. 2023), and natricine snakes (Coppinger et al. 2025) suggest that substantial diversity remains unexplored. Understanding the ecological and genetic basis of individual-level venom variation will be essential for linking selection at fine spatial and temporal scales to broader patterns of venom evolution.

### Sexual dimorphism

Empirical evidence for intersexual venom variation has historically been limited and inconsistent. Early studies generally reported little to no venom differences between sexes (Chippaux et al. 1991), suggesting broad conservation of venom composition. However, some biochemical studies hinted at potential sexual differences. An isoelectric focusing analysis of *Calloselasma rhodostoma* venoms detected a single unidentified protein band present in females but absent in males (Daltry et al. 1996). More recent proteomic studies have revealed subtle sex-associated differences in some species. In *Bothrops jararaca*, sex-specific protein bands were detected using one-dimensional electrophoresis, spot intensities differed on two-dimensional gels, and enzyme activities varied between sexes (Menezes et al. 2006; Pimenta et al. 2007). A MALDI-TOF mass spectrometry analysis further identified four bradykinin-potentiating peptides present exclusively in female venoms, although substantial individual variation and unclear functional significance complicate interpretation (Pimenta et al. 2007). Additional studies reported sexual differences in proteolytic activity toward fibrinogen and gelatin (Zelanis et al. 2016), as well as sex-specific variation in enzymatic and toxic activities (Furtado et al. 2006). An analysis of neonate *Bothrops moojeni* likewise found higher serine protease effects, hemorrhagic activity, and lethality in female venoms relative to males (Ferreira-Rodrigues et al. 2024). However, other studies have reported no sex-based venom differences in dimorphic pit viper species (Da Silva Aguiar et al. 2020; Gómez et al. 2021), including *Tropidolaemus wagleri* (Tan et al. 2017), despite its extreme sexual size dimorphism.

Sex-associated venom differences have also been reported in elapid snakes. In *Naja atra*, male and female venoms differed in acetylcholinesterase, nerve growth factor, and CRISPs based on electrophoretic and enzymatic analyses (Nie et al. 2022). In contrast, a recent integrative proteomic study found no evidence of sexual dimorphism in *Vipera berus* venoms (Schulte et al. 2026a). Overall, sexual dimorphism in venom composition appears subtle, species-specific, and far from universal. The ecological and evolutionary mechanisms underlying such variation—including sex-specific diets, reproductive roles, defensive behaviors, or hormonal regulation—remain poorly understood. Targeted, hypothesis-driven studies will be required to assess the prevalence and adaptive significance of venom sexual dimorphism across snakes.

### Geographic variation

Although venom composition is relatively conserved in some snake species (Avella et al. 2023; Balchan et al. 2025b), geographically structured variation is widespread and represents the dominant pattern across snakes. Early investigations focused on the Mohave rattlesnake (*Crotalus scutulatus*), a species exhibiting multiple venom phenotypes with markedly different lethality. Initial work established the existence of these phenotypes (Glenn et al. 1983), and subsequent studies resolved their geographic structuring and environmental correlates (Zancolli et al. 2016; Strickland et al. 2018). Extensive geographic variation has since been documented in numerous rattlesnake species (Sunagar et al. 2014; Margres et al. 2021; Smith et al. 2023a), with geographically structured venom phenotypes representing a recurring pattern within the group (Mackessy 2008, 2010b).

Beyond rattlesnakes, geographic venom variation has been documented in other pit vipers (Crotalinae; Mora-Obando et al. 2020) and true vipers (Viperinae; Damm et al. 2024; Sarangi et al. 2025). Although fewer studies exist for elapid snakes, pronounced geographic variation has long been recognized in Asian cobras (*Naja* spp.; Chanda et al. 2018; Rashmi et al. 2021; Deka et al. 2023) and kraits (*Bungarus* spp.; Oh et al. 2019; Sunagar et al. 2021), where venom heterogeneity has important snakebite implications (Sintiprungrat et al. 2016). More recent studies have documented geographic variation in Australian (Hydrophiinae; Van Thiel et al. 2023), African (Elapinae; Hus et al. 2024), and Asian elapids (Elapinae; Tan et al. 2015). Geographic venom variation remains poorly studied in colubrid snakes and *helodermatid* lizards. Existing evidence suggests substantial variation in colubrine (Mackessy et al. 2006), dipsadine (Tioyama et al. 2023), and natricine snakes (Coppinger et al. 2025), whereas *Heloderma* venoms may be more conserved (Koludarov et al. 2014).

Recent work also highlights the potential role of abiotic environmental variables in shaping venom phenotypes (Strickland et al. 2018; Smith et al. 2023a; Sarangi et al. 2025). Although geographic patterns of venom variation are now well described for many species, the drivers underlying these patterns remain poorly resolved outside a few model systems. Identifying the biotic and abiotic forces structuring venom variation across landscapes remains a key frontier for understanding the eco-evolutionary dynamics of venom systems.

### Ontogenetic variation

Ontogenetic changes in venom composition are widespread and often align with ecological and body size shifts across an individual’s life history. Changes in habitat use, prey size, prey type, and predator exposure across developmental stages can impose stage-specific selective pressures on venom function (e.g., Mackessy et al. 2003). Ontogenetic venom variation is well documented in rattlesnakes (Mackessy 1988; Saviola et al. 2015; Borja et al. 2018; Mackessy et al. 2018) and other New World pit vipers (Guércio et al. 2006; Alape-Girón et al. 2008; Barlow et al. 2009; Machado Braga et al. 2020), where juveniles and adults often exhibit distinct venom compositions reflecting shifts from ectotherm- to endotherm-based diets. Two common trends include higher toxicity and reduced metalloproteinase content in neonate venoms relative to adults. These patterns likely reflect ontogenetic changes in prey communities (e.g., Andrade and Abe 1999), with venoms optimized for prey targeted at specific life stages. Comparable ontogenetic patterns occur in true vipers, including *Daboia* (Zdenek et al. 2022; Senji Laxme et al. 2024), *Echis* (Barker et al. 2025), and *Vipera* (Avella et al. 2022a; Qiao et al. 2024; Lakušić et al. 2025, 2025), though venom ontogeny remains unevenly studied across the group. Alternative patterns also occur; for example, juvenile *Vipera* venoms are often richer in SVMPs than those of adults (Schulte et al. 2026b; Avella et al. 2022a). In elapids, evidence for ontogenetic venom variation is mixed. Venoms of *Naja naja* appear largely consistent across developmental stages (Senji Laxme et al. 2024), whereas *Naja kaouthia* shows age-related differences in PLA₂s activity and isoforms (Modahl et al. 2016). In the Australian genus *Pseudonaja*, most species transition from noncoagulopathic venoms in juveniles to coagulopathic venoms in adults, likely reflecting shifts from reptilian to mammalian diets (Cipriani et al. 2017). Ontogenetic variation remains largely unexplored in helodermatid lizards (Dobson et al. 2024) and colubrid snakes (Mackessy et al. 2006), where diet and trophic ecology are poorly known for most species.

### Seasonal variation

Because prey availability can change throughout the year, seasonality has been proposed as a potential driver of venom variation (Chippaux et al. 1991), although empirical support remains limited. A study on several rattlesnake species (*Crotalus atrox, C. molossus*, and *C. oreganus helleri*) detected no seasonal differences in venom composition over extended sampling periods (Gregory-Dwyer et al. 1986). Similarly, studies on *C. durissus* and *C. molossus* found no compositional changes but did detect seasonal variation in toxin activity (Macías-Rodríguez et al. 2014; Tasima et al. 2024). Early work suggested seasonal differences in *Vipera ammodytes*, with certain protein bands present in summer but absent in winter electrophoretic profiles (Gubenšek et al. 1974). However, venomic profiling of *Pseudonaja textilis* sampled over a 12-month period found no evidence of seasonal variation (Williams and White 1992). Overall, seasonal venom variation appears weakly supported and may be subtle, context-dependent, or expressed primarily at the level of toxin activity rather than composition. Future work may benefit from focusing on species inhabiting strongly seasonal environments, where fluctuating ecological conditions could impose differential selection on venom phenotypes.

### Interspecific variation

A general expectation is that closely related taxa possess more similar venoms than phylogenetically distant relatives. At broad scales, the dominance and relative abundance of major toxin families follow clear phylogenetic patterns (Mackessy 2010a, 2021). Viperid venoms are typically dominated by higher-molecular-weight enzymatic toxins such as SVMPs and serine proteases (SVSPs), whereas elapid venoms are largely composed of smaller, nonenzymatic toxins, particularly three-finger toxins (3FTxs) and PLA₂s (Tasoulis and Isbister 2017; Barua and Mikheyev 2019). Colubrid snakes exhibit diverse venom phenotypes, most still poorly characterized, though several are rich in 3FTxs (Mackessy 2002; Junqueira-de-Azevedo et al. 2016; Modahl and Mackessy 2019).

Phylogenetic components of venom variation have been examined in several viperid (Mackessy 2010b; Gibbs et al. 2013; Holding et al. 2021; Zhao et al. 2023) and elapid clades (Cipriani et al. 2017; Sanz et al. 2019; Kazandjian et al. 2021; Van Thiel et al. 2023). Although these studies link clade-level venom differences to ecological drivers such as diet (Holding et al. 2021) and defense (Kazandjian et al. 2021), much of the phylogenetic structure underlying venom evolution remains unresolved. Despite expectations of phylogenetic signal, venom phenotypes often do not track evolutionary relationships within clades. A well-known example is the western rattlesnake complex (*Crotalus viridis/C. oreganus*), which exhibits two divergent venom phenotypes: a widespread proteolytic Type I venom, presumed ancestral, and a highly toxic Type II venom that has evolved repeatedly within the group (Mackessy 2008, 2010b). These phenotypes do not correspond to phylogenetic relationships and instead appear to have arisen multiple times independently. Similar patterns occur broadly across rattlesnakes (*Crotalus* spp.), where toxic phenotypes repeatedly evolve within clades otherwise dominated by proteolytic venoms (Colis-Torres et al. 2022; Borja et al. 2025).

In elapids, a PLA₂/3FTx dichotomy has been described in *Micrurus* coral snakes (Sanz et al. 2016, 2019) and Australo-Papuan elapids (Goldenberg et al. 2018; Van Thiel et al. 2023), where venoms are typically dominated by one of these toxin classes. At broader taxonomic scales, similar trade-offs between enzyme-rich and neurotoxin-dominated venoms may occur across venomous snakes, with venom proteomes tending toward either enzymatic or PLA₂/3FTx-dominated compositions (Barua and Mikheyev 2019). These patterns suggest ecological pressures may override phylogenetic inertia in shaping venom phenotypes. In contrast, venoms of *Heloderma* lizards appear highly conserved across phylogeny (Koludarov et al. 2014), possibly reflecting ecological similarity among species and a shared defensive function (Russell and Bogert 1981), although limited ecological data preclude firm conclusions.

Interpretations of interspecific venom variation may also be complicated by discordance between venom gland transcriptomes and expressed proteomes (Sunagar et al. 2021), which may, for example, be driven by post-transcriptional regulation (Rokyta et al. 2015), alternative splicing (Ogawa et al. 2019), and/or post-translational modifications (Andrade-Silva et al. 2016). For example, genes encoding 3FTxs have been detected in venom gland transcriptomes (Junqueira-de-Azevedo et al. 2006; Pahari et al. 2007) despite the absence of corresponding toxins in venom proteomes from the same species (Sanz et al. 2006). Such incongruence highlights the importance of integrating transcriptomic, proteomic, and ecological data when assessing phylogenetic patterns of venom evolution (Davies and Arbuckle 2019; Schaeffer et al. 2023; Zhao et al. 2023; Modahl et al. 2024; Heptinstall et al. 2025).

### Hybridization-induced variation

Hybridization is increasingly recognized as a contributor to phenotypic diversity across taxa (e.g., Myers et al. 2024), including venom phenotypes. Among venomous snakes, hybridization occurs both as isolated events (Neri-Castro et al. 2022) and within stable hybrid zones between species (Zancolli et al. 2016; Smith et al. 2023b). Laboratory studies show that interspecific hybridization can produce venom phenotypes intermediate to those of parental species (Aird et al. 1989; Smith and Mackessy 2016), and similar patterns occur in wild populations. Hybridization between the native *Protobothrops flavoviridis* and introduced *P. elegans* in the Ryukyu Islands produced individuals with intermediate venom phenotypes (Aird et al. 2015). Comparable results have been reported in several rattlesnake hybrid systems (Harrison et al. 2022; Neri-Castro et al. 2022; Roldán-Padrón et al. 2022; Smith et al. 2023b).

A prominent example occurs in a stable hybrid zone between *Crotalus viridis* and *C. scutulatus* in southwestern New Mexico, USA. *C. viridis* expresses a proteolytic Type I venom, whereas *C. scutulatus* produces a Type II venom characterized by the presynaptically neurotoxic PLA₂ Mojave toxin. Although pure *C. scutulatus* and admixed individuals within the contact zone possess Mojave toxin, it is absent from pure *C. viridis* populations outside the zone (Zancolli et al. 2016). This pattern suggests that hybridization can transfer potent toxins across species boundaries, while natural selection may constrain their introgression despite substantial gene flow.

### Convergent evolution

Convergent evolution arises when similar selective pressures act on distantly related taxa and may occur through morphological (Muschick et al. 2012), behavioral (Blackledge and Gillespie 2004), or molecular mechanisms (Van Thiel et al. 2022). Although a wide diversity of toxins has evolved across the tree of life, there are relatively few biochemical pathways through which venoms incapacitate prey (e.g., membrane disruption or inhibition of neurotransmission). Consequently, striking cases of convergence among independently evolved or recruited toxins have been documented (Casewell et al. 2013). As a molecular component of the organismal phenotype, venom is therefore expected to exhibit convergent evolution under shared ecological pressures. However, few studies have explicitly examined venom convergence alongside convergent evolution in ecology, morphology, or behavior.

At broader scales, convergent patterns of toxin composition and abundance occur across diverse snake lineages. Venom proteomes frequently trend toward either PLA₂/3FTx-dominated or enzyme-rich configurations (Barua and Mikheyev 2019), suggesting similar functional demands may repeatedly shape venom phenotypes independent of shared ancestry.

Promising systems for investigating venom convergence include distantly related species with similar dietary specialization, as well as taxa that have independently transitioned into comparable ecological niches such as aquatic, arboreal, or fossorial habitats. Comparative analyses across these systems may help disentangle the relative roles of ecology and phylogeny in shaping venom evolution.

### Prey specificity

Physiological differences among prey taxa impose distinct functional requirements on venom, favoring toxins optimized for subjugating specific prey types (Daltry et al. 1996). Diet was among the first ecological factors identified as a driver of venom variation, and regional differences in prey availability can exert strong selective pressures on venom proteomes.

Prey community variation drives venom divergence across numerous species (Daltry et al. 1996; Smith et al. 2023a) and appears widespread. In species with strong prey specificity, venoms may include taxon-specific toxins. Notable examples in colubrids include the bird-specific irditoxins A and B from *Boiga irregularis* (Pawlak et al. 2009), the lizard-specific fulgimotoxin from *Oxybelis fulgidus* (Heyborne and Mackessy 2021), and the lizard-specific sulditoxin and mammal-specific sulmotoxin 1 from *Spilotes sulphureus* (Modahl et al. 2018), all specialized 3FTxs. Among vipers, prey-specific toxins are formally characterized only in *Crotalus viridis*, where myotoxin a targets mammalian prey (Smith et al. 2023; Balchan et al. 2024). However, many vipers show prey-specific effects inferred from differential lethality across prey classes (Gibbs and Mackessy 2009; Richards et al. 2012; Healy et al. 2019; Lyons et al. 2020), suggesting prey specialization may be more widespread than currently documented, and underscoring a need for continued research.

### Venom resistance

Prey species may experience strong selection to evolve physiological mechanisms that neutralize the venoms of their predators (Holding et al. 2016a, 2016b). This has been intensively studied in snake–rodent systems, notably the California ground squirrel (*Otospermophilus beecheyi*) and Pacific rattlesnake (*Crotalus oreganus*). Beyond behavioral defenses (Rundus et al. 2007), squirrels possess biochemical mechanisms for detoxifying rattlesnake venoms (Poran et al. 1987; Robinson et al. 2021). Resistance varies geographically in this system, highlighting its evolutionary lability.

Venom resistance also occurs in numerous other snake–prey systems (Heatwole and Powell 1998; Chandrasekara et al. 2024) across North America (Pomento et al. 2016; Robinson et al. 2021; Balchan et al. 2024), South America (Voss 2013), Africa (Phillips et al. 2012), and Australia (Chandrasekara et al. 2024, 2025). The taxonomic and geographic breadth of resistance emphasizes the role of reciprocal selection and suggests coevolutionary arms races as key drivers of venom diversification.

Predators that frequently consume venomous snakes also show selection for resistance, including opossums (Didelphidae; Jansa and Voss 2011; Voss and Jansa 2012), mongooses (Herpestidae; Bdolah et al. 1997), and honey badgers (*Mellivora capensis*; Drabeck et al. 2015). However, relatively few studies have examined resistance in these predators, highlighting an important gap and a promising avenue for future research.

## Methods and mindsets: Past, present and future

**Fig. 3:**
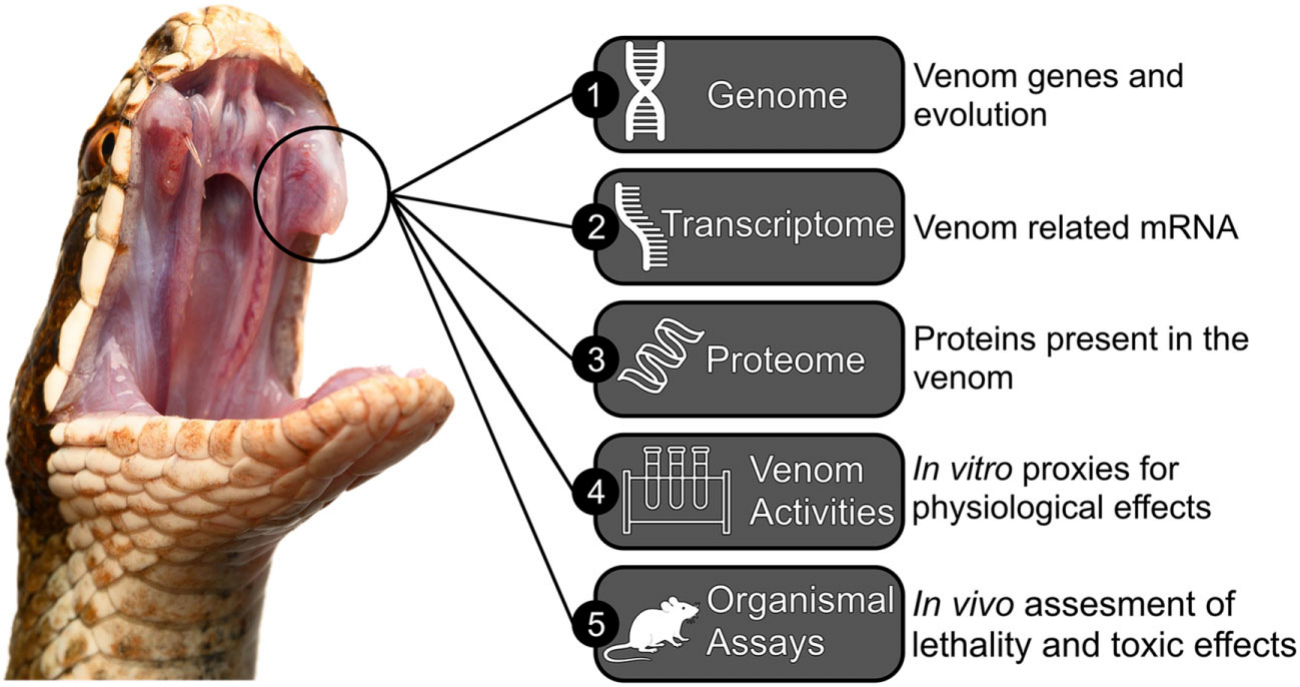
Conceptual representation of the layered methods of studying venom variation. The five layers described here may be used in venomic investigations either singly or in combination and may address questions at different levels. Bridging these layers (e.g., via proteogenomics) may offer novel insights requiring integration across levels and datasets. Photo of *Vipera berus* by Thor Håkonsen and used with permission.

Research questions in venom biology have evolved over time, as have the methods used to address them. These methods target multiple levels of biological organization (Fig. 3), each providing distinct insights into venom complexity. Integrating data across levels is essential for linking genotype, phenotype, and extended phenotype, enabling a more comprehensive understanding of venom function and evolution.

**Fig. 4:**
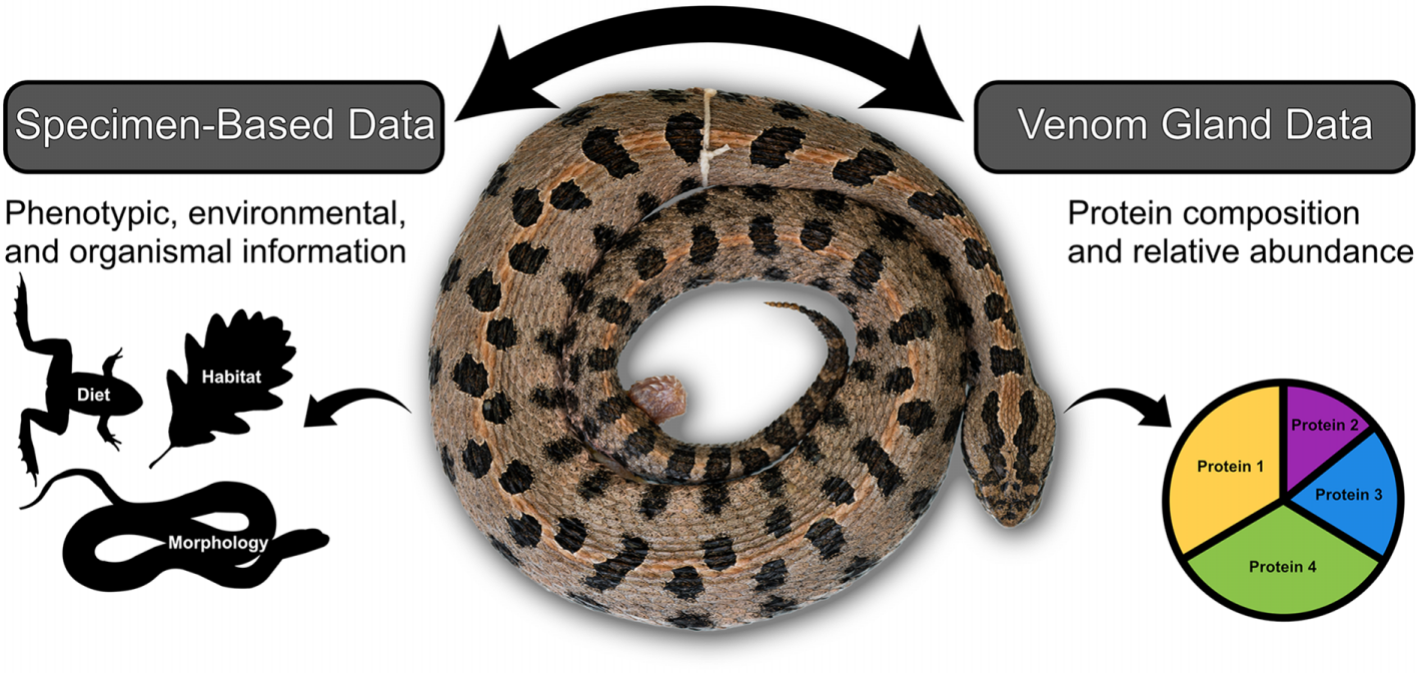
The natural history specimen represents a largely untapped resource from which to study venom variation. In addition to phenotypic data that may be pulled from the organism itself, the preserved venom gland represents a source of venom that may be used in addressing questions with geographic or temporal themes. Photo of *Sistrurus miliarius* specimen taken by Owen M. Edwards and used with permission.

### Past

The earliest experimental studies of snake venom, beginning in the seventeenth century, were largely exploratory. Redi (1664) confirmed venom toxicity using whole-organism assays, and Fontana (1781) examined potency, dose dependence, and physiological effects. These studies advanced understanding of venom effects but treated venom as a static substance rather than a dynamic trait shaped by ecology or evolution.

In the nineteenth and early twentieth centuries, methodological advances allowed more detailed characterization of venom composition. Researchers catalogued components, quantified relative abundances, and contextualized venom systems. Major toxin families were first described during this period: PLA₂s (“hemolysins”) in the 1940s (De 1944), SVSPs (“reptilase”) in the 1950s (Bruck and Salem 1954), 3FTxs in the 1960s (Chang and Lee 1963), and SVMPs in the 1990s (Bjarnason and Fox 1994).

As resolution improved, venoms were shown to vary within species. Individual-level variation (Jímenez-Porras 1964; Glenn and Straight 1977), population differences (Aragón and Gubenšek 1981), and geographic structuring of venom phenotypes (Glenn and Straight 1978; Glenn et al. 1983; Minton and Weinstein 1986) were documented. Hybridization and introgression were shown to reshape venom phenotypes using immunochemical and functional assays, lethality tests, electrophoresis, and HPLC (Glenn and Straight 1989, 1990).

Parallel work on venom resistance demonstrated that prey could evolve physiological mechanisms to neutralize venoms. Rodents (Perez et al. 1978a, 1978b; 1979; De Wit 1982; Poran et al. 1987), opossums (Kilmon 1976; Werner and Vick 1977), and other taxa (Bdolah et al. 1997; Heatwole et al. 1999) were shown to detoxify venom using whole-organism and inhibition-based assays, measuring median lethal doses and venom neutralization capacities of various species. Diet also emerged as a major driver of venom evolution (Daltry et al. 1996), reinforced by prey-specific susceptibility (Jorge da Silva and Aird 2001) and ontogenetic shifts in diet and venom composition (Mackessy 1988).

Despite methodological and research biases, such as taxonomic skews toward viperids, geographic skews for Neotropical taxa, and an absence of integrated ecological data (Avella et al. 2022b), these early studies established enduring themes in venom research—variation, adaptation, and ecological interaction—while leaving the mechanisms largely unresolved.

### Present

Contemporary research has transformed venoms from descriptive biochemical curiosities into tractable model systems for studying eco-evolutionary dynamics. This shift reflects both conceptual advances and analytical innovations that allow venoms to be interrogated at unprecedented resolution.

A major inflection point came with mass spectrometry-based analyses and the rise of the venomics framework in the early 2000s (Juárez et al. 2004; Calvete et al. 2007; Gutiérrez et al. 2009). By integrating reversed-phase high-performance liquid chromatography, gel electrophoresis, and mass spectrometry, venomics enabled comprehensive quantification of entire venom proteomes, reframing venoms as integrated systems rather than incomplete toxin inventories. Advances in liquid chromatography–tandem mass spectrometry and bioinformatic pipelines facilitated peptide-based identification from crude venoms, popularizing shotgun proteomics (OmPraba et al. 2010; Tan et al. 2016; Kunalan et al. 2018; Koua et al. 2022). Combined with traditional techniques such as gel electrophoresis, toxicity assays, and enzymatic activity assays (Lüddecke et al. 2024), these methods now permit fine-scale comparisons of venom composition, abundance, and function. Sequencing technologies have produced species-specific omics databases, including venom gland transcriptomes and genomes, allowing venom systems to be studied at the level of gene expression and regulatory architecture (Pahari et al. 2007; Casewell et al. 2009; Walker et al. 2020; Slagboom et al. 2022; Avella et al. 2024, 2025).

These methodological advances have coincided with a shift toward explicitly ecological and evolutionary questions. Venom datasets are increasingly integrated with phylogenetic (Holding et al. 2021; Kazandjian et al. 2021), environmental (Sarangi et al. 2025), and geographic data (Strickland et al. 2018; Avella et al. 2023; Smith et al. 2023). Studies of venom resistance now explore community-level dynamics (Robinson et al. 2021; Balchan et al. 2024, 2025a) and underlying molecular mechanisms (Jansa and Voss 2011; Gibbs, et al. 2020)

Ontogenetic venom variation is routinely examined across life stages (Mackessy et al. 2018), being increasingly contextualized within ecological and environmental frameworks (Mackessy et al. 2003; Lakušić et al. 2025). Individual-level variation within populations is now recognized as biologically meaningful, influencing foraging and prey capture (Smiley-Walters et al. 2019; Rashmi et al. 2021). Growing attention to predator–prey interactions continues to generate novel hypotheses about venom evolution, driving the development of new experimental and analytical approaches (Tian et al. 2020; Holding et al. 2021).

### Future

Despite methodological and conceptual advances, the study of venom variation remains ripe for expansion. Future work should prioritize explicit tests of selective pressures, moving beyond correlations to experimentally assess how diet, prey physiology, or environmental factors shape venom phenotypes. Integrating molecular, organismal, and ecological data remains critical, with functional assays, reciprocal prey tests, and field manipulations linking venom composition to ecological performance. Combining genomics, transcriptomics, proteomics, functional assays, and field ecology can connect genotype, phenotype, and fitness, revealing mechanisms driving venom diversification (Drukewitz and Von Reumont 2019; Calvete et al. 2021; Lüddecke et al. 2023). Data sharing and centralized platforms will further accelerate these efforts (Zancolli et al. 2024; Castoe et al. 2025; Mehr et al. 2026).

Expanding taxonomic coverage beyond well-studied or medically relevant groups—particularly colubrids and helodermatid lizards—will help distinguish general patterns from lineage-specific traits. Fine-scale variation, including individual, sex-specific, seasonal, and microgeographic differences, warrants closer attention due to potential ecological and fitness consequences. Incorporating coevolutionary dynamics, such as venom resistance in prey and predators, will clarify reciprocal selection pressures shaping venom evolution.

Finally, venom systems provide a lens to study responses to rapid environmental change. Museum collections hold hundreds of thousands of venomous snake specimens (Fig. 4) spanning the past two centuries. Although venom in these preserved glands is largely denatured, protein identification and quantification may still be possible (Esquerré et al. 2026), enabling investigations of temporal and geographic variation otherwise inaccessible.

## Conclusions

Snake venoms are dynamic, multifunctional traits shaped by a network of ecological and evolutionary pressures. Variation occurs across multiple scales—geographic, individual, ontogenetic, seasonal, interspecific, and sex-specific—reflecting interactions among diet, prey resistance, hybridization, and convergent adaptation. While phylogeny provides a framework for broad toxin patterns, ecological forces often override evolutionary inertia, generating remarkable phenotypic diversity. Advances in proteomics, transcriptomics, functional assays, and integrative field studies now allow direct links between venom composition, ecological performance, and evolutionary outcomes.

As complex traits, snake venoms offer an exceptional system for studying eco-evolutionary processes. Integrating existing and emerging methods with explicit ecological and evolutionary questions promises deeper insights into venom phenotypes. Expanding taxonomic breadth, incorporating fine-scale and coevolutionary dynamics, and leveraging new data sources will be critical for uncovering the drivers of venom diversification. Ultimately, venoms illustrate how selection, constraint, and ecological context interact to shape phenotypes across time and space.

## Data Availability

No new data were generated or analysed in support of this research.

## References

[bib1] Aird SD, Aggarwal S, Villar-Briones A, Tin MM-Y, Terada K, Mikheyev AS. 2015. Snake venoms are integrated systems, but abundant venom proteins evolve more rapidly. BMC Genomics. 16:647. 26315097 10.1186/s12864-015-1832-6 PMC4552096

[bib2] Aird SD, Thirkhill LJ, Seebart CS, Kaiser II. 1989. Venoms and morphology of western diamondback/Mojave rattlesnake hybrids. J Herpetol. 23:131–41.

[bib3] Alape-Girón A, Sanz L, Escolano J, Flores-Díaz M, Madrigal M, Sasa M, Calvete JJ. 2008. Snake venomics of the lancehead pitviper *Bothrops asper*: geographic, individual, and ontogenetic variations. J Proteome Res. 7:3556–71. 18557640 10.1021/pr800332p

[bib4] Almeida GDO, Oliveira ISD, Arantes EC, Sampaio SV. 2023. Snake venom disintegrins update: insights about new findings. J Venom Anim Toxins incl Trop Dis. 29:e20230039. 37818211 10.1590/1678-9199-JVATITD-2023-0039 PMC10561651

[bib5] Andrade DV, Abe AS. 1999. Relationship of venom ontogeny and diet in *Bothrops*. Herpetologica. 55:200–4.

[bib6] Andrade-Silva D, Zelanis A, Kitano ES, Junqueira-de-Azevedo ILM, Reis MS, Lopes AS, Serrano SMT. 2016. Proteomic and glycoproteomic profilings reveal that post-translational modifications of toxins contribute to venom phenotype in snakes. J Proteome Res. 15:2658–75. 27297130 10.1021/acs.jproteome.6b00217

[bib7] Ang WF, Koh CY, Kini RM. 2022. From snake venoms to therapeutics: a focus on natriuretic peptides. Pharmaceuticals. 15:1153. 36145374 10.3390/ph15091153 PMC9502559

[bib8] Aragón F, Gubenšek F. 1981. Bothrops asper venom from the Atlantic and Pacific zones of Costa Rica. Toxicon. 19:797–805. 7038989 10.1016/0041-0101(81)90076-3

[bib9] Arbuckle K . 2015. Evolutionary context of venom in animals. In: Gopalakrishnakone P, Malhotra A, editors. Evolution of venomous animals and their toxins. Dordrecht: Springer Netherlands. p.1–23.

[bib10] Arlinghaus FT, Eble JA. 2012. C-type lectin-like proteins from snake venoms. Toxicon. 60:512–9. 22781131 10.1016/j.toxicon.2012.03.001

[bib11] Avella I, Calvete JJ, Sanz L, Wüster W, Licata F, Quesada-Bernat S, Rodríguez Y, Martínez-Freiría F. 2022a. Interpopulational variation and ontogenetic shift in the venom composition of Lataste’s viper (*Vipera latastei*, Boscá 1878) from northern Portugal. J Proteomics. 263:104613. 35589061 10.1016/j.jprot.2022.104613

[bib12] Avella I, Wüster W, Luiselli L, Martínez-Freiría F. 2022b. Toxic habits: an analysis of general trends and biases in snake venom research. Toxins. 14:884. 36548781 10.3390/toxins14120884 PMC9783912

[bib13] Avella I, Damm M, Freitas I, Wüster W, Lucchini N, Zuazo Ó, Süssmuth RD, Martínez-Freiría F. 2023. One size fits all—Venomics of the Iberian adder (*Vipera seoanei*, Lataste 1878) reveals low levels of venom variation across its distributional range. Toxins. 15:371. 37368672 10.3390/toxins15060371 PMC10301717

[bib14] Avella I, Schulte L, Damm M, Uhrig L, Cabrera-Orefice A, Eichberg J, Hardes K, Hurka S, Lindner T, Vilcinskas A et al. 2025. Venomics of the Arabian saw-scaled viper (*Echis coloratus*) through transcriptome-guided proteomics and in vitro functional profiling. PLoS Negl Trop Dis. 19:e0013439. 40811711 10.1371/journal.pntd.0013439 PMC12373283

[bib15] Avella I, Schulte L, Hurka S, Damm M, Eichberg J, Schiffmann S, Henke M, Timm T, Lochnit G, Hardes K et al. 2024. Proteogenomics-guided functional venomics resolves the toxin arsenal and activity of *Deinagkistrodon acutus* venom. Int J Biol Macromol. 278:135041. 39182889 10.1016/j.ijbiomac.2024.135041

[bib16] Balchan NR, Crowther TW, Kratz G, Mackessy SP. 2025a. Raptors without resistance: no evidence for endogenous inhibition of rattlesnake venom metalloproteinases in a Great Plains raptor assemblage. Toxicon. 256:108275. 39914593 10.1016/j.toxicon.2025.108275

[bib17] Balchan NR, Vick CP, Mackessy SP. 2025b. Western diamondback rattlesnake (*Crotalus atrox*) venoms show increased snake venom metalloproteinase abundance and activity at their northeastern distributional limits. Toxicon. 265:108497. 40675498 10.1016/j.toxicon.2025.108497

[bib18] Balchan NR, Smith CF, Mackessy SP. 2024. A plethora of rodents: rattlesnake predators generate unanticipated patterns of venom resistance in a grassland ecosystem. Toxicon: X. 21:100179. 38144228 10.1016/j.toxcx.2023.100179 PMC10746501

[bib19] Barker A, Jones L, Bourke LA, Seneci L, Chowdhury A, Violette A, Fourmy R, Soria R, Aldridge M, Fry BG. 2025. Snake venom makeover: age-dependent variations in procoagulant biochemistry of Egyptian saw-scaled viper (*Echis pyramidum pyramidum*) venom. Toxins. 17:149. 40137922 10.3390/toxins17030149 PMC11946080

[bib20] Barlow A, Pook CE, Harrison RA, Wüster W. 2009. Coevolution of diet and prey-specific venom activity supports the role of selection in snake venom evolution. Proc. R. Soc. B. 276:2443–9. 10.1098/rspb.2009.0048 PMC2690460 19364745

[bib21] Barua A, Mikheyev AS. 2019. Many options, few solutions: over 60 my snakes converged on a few optimal venom formulations. Mol Biol Evol. 36:1964–74. 31220860 10.1093/molbev/msz125 PMC6736290

[bib22] Bdolah A, Kochva E, Ovadia M, Kinamon S, Wollberg Z. 1997. Resistance of the Egyptian mongoose to sarafotoxins. Toxicon. 35:1251–61. 9278974 10.1016/s0041-0101(97)00019-6

[bib23] Beck DD . 2005. Biology of gila monsters and beaded lizards. Berkley, CA: University of California Press

[bib24] Bernheimer AW, Linder R, Weinstein SA, Kim K-S. 1987. Isolation and characterization of a phospholipase B from venom of Collett's snake, *Pseudechis colletti*. Toxicon. 25:547–54. 3617089 10.1016/0041-0101(87)90290-x

[bib25] Bjarnason JB, Fox JW. 1994. Hemorrhagic metalloproteinases from snake venoms. Pharmacol Ther. 62:325–72. 7972338 10.1016/0163-7258(94)90049-3

[bib26] Blackledge TA, Gillespie RG. 2004. Convergent evolution of behavior in an adaptive radiation of Hawaiian web-building spiders. Proc Natl Acad Sci USA. 101:16228–33. 15520386 10.1073/pnas.0407395101 PMC528981

[bib27] Borja M, Castañeda-Gaytán G, Alagón A, Strickland JL, Parkinson CL, Gutiérrez-Martínez A, Rodriguez-López B, Zarzosa V, Lomonte B, Saviola AJ et al. 2025. Venom variation and ontogenetic changes in the *Crotalus molossus* complex: insights into composition, activities, and antivenom neutralization. Comp Biochem Physiol C Toxicol Pharmacol. 290:110129. 39892555 10.1016/j.cbpc.2025.110129

[bib28] Borja M, Neri-Castro E, Pérez-Morales R, Strickland JL, Ponce-López R, Parkinson CL, Espinosa-Fematt J, Sáenz-Mata J, Flores-Martínez E, Alagón A et al. 2018. Ontogenetic change in the venom of Mexican black-tailed rattlesnakes (*Crotalus molossus nigrescens*). Toxins. 10:501. 30513722 10.3390/toxins10120501 PMC6315878

[bib29] Bruck H, Salem G. 1954. Reptilase, a hemostatic for prophylaxis and therapy in surgical operations. Wiener Klin Wochenschr. 66:395–7. 13187962

[bib30] Calvete JJ . 2013. The continuing saga of snake venom disintegrins. Toxicon. 62:40–9. 23010163 10.1016/j.toxicon.2012.09.005

[bib31] Calvete JJ, Juárez P, Sanz L. 2007. Snake venomics. Strategy and applications. J Mass Spectrom. 42:1405–14. 17621391 10.1002/jms.1242

[bib32] Calvete JJ, Lomonte B, Saviola AJ, Bonilla F, Sasa M, Williams DJ, Undheim EAB, Sunagar K, Jackson TNW. 2021. Mutual enlightenment: a toolbox of concepts and methods for integrating evolutionary and clinical toxinology via snake venomics and the contextual stance. Toxicon. 9–10:100070. 10.1016/j.toxcx.2021.100070 PMC8234350 34195606

[bib33] Calvete JJ, Marcinkiewicz C, Monleón D, Esteve V, Celda B, Juárez P, Sanz L. 2005. Snake venom disintegrins: evolution of structure and function. Toxicon. 45:1063–74. 15922775 10.1016/j.toxicon.2005.02.024

[bib34] Casewell NR, Harrison RA, Wüster W, Wagstaff SC. 2009. Comparative venom gland transcriptome surveys of the saw-scaled vipers (Viperidae: *Echis*) reveal substantial intra-family gene diversity and novel venom transcripts. BMC Genomics. 10:564. 19948012 10.1186/1471-2164-10-564 PMC2790475

[bib35] Casewell NR, Jackson TNW, Laustsen AH, Sunagar K. 2020. Causes and consequences of Snake venom variation. Trends Pharmacol Sci. 41:570–81. 32564899 10.1016/j.tips.2020.05.006 PMC7116101

[bib36] Casewell NR, Wagstaff SC, Harrison RA, Renjifo C, Wuster W. 2011. Domain loss facilitates accelerated evolution and neofunctionalization of duplicate snake venom metalloproteinase toxin genes. Mol Biol Evol. 28:2637–49. 21478373 10.1093/molbev/msr091

[bib37] Casewell NR, Wüster W, Vonk FJ, Harrison RA, Fry BG. 2013. Complex cocktails: the evolutionary novelty of venoms. Trends Ecol Evol. 28:219–29. 23219381 10.1016/j.tree.2012.10.020

[bib38] Castoe TA, Daly M, Jungo F, Kirchhoff KN, Koludarov I, Mackessy S, Macrander J, Mehr S, Modica MV, Sanchez EE et al. 2025. A vision for VenomsBase: an integrated knowledgebase for the study of venoms and their applications. Integr Organ Biol. 7:obaf026. 1–14. 10.1093/iob/obaf026 PMC12259279 40661153

[bib39] Chan YS, Cheung RCF, Xia L, Wong JH, Ng TB, Chan WY. 2016. Snake venom toxins: toxicity and medicinal applications. Appl Microbiol Biotechnol. 100:6165–81. 27245678 10.1007/s00253-016-7610-9

[bib40] Chanda A, Patra A, Kalita B, Mukherjee AK. 2018. Proteomics analysis to compare the venom composition between *Naja naja* and *Naja kaouthia* from the same geographical location of eastern India: correlation with pathophysiology of envenomation and immunological cross-reactivity towards commercial polyantivenom. Expert Rev Proteomics. 15:949–61. 30345852 10.1080/14789450.2018.1538799

[bib41] Chandrasekara U, Broussard EM, Rokyta DR, Fry BG. 2024. High-voltage toxin’roll: electrostatic charge repulsion as a dynamic venom resistance trait in pythonid snakes. Toxins. 16:176. 38668601 10.3390/toxins16040176 PMC11053703

[bib42] Chandrasekara U, Mancuso M, Seneci L, Bourke L, Trembath DF, Sumner J, Zdenek CN, 2024. A Russian doll of resistance: nested gains and losses of venom immunity in varanid lizards. IJMS. 25:2628. 38473875 10.3390/ijms25052628 PMC10932397

[bib43] Chandrasekara U, Mancuso M, Shea G, Jones L, Kwiatkowski J, Trembath D, Chowdhury A, Bertozzi T, Gardner MG, Hoskin CJ et al. 2025. Make acetylcholine great again! Australian skinks evolved multiple neurotoxin-proof nicotinic acetylcholine receptors in defiance of snake venom. IJMS. 26:7510. 40806638 10.3390/ijms26157510 PMC12347452

[bib44] Chang CC, Lee CY. 1963. Isolation of neurotoxins from the venom of *Bungarus multicinctus* and their modes of neuromuscular blocking action. Arch Int Pharmacodyn Ther. 144:241–57. 14043649

[bib45] Chang D, Duda TF. 2012. Extensive and continuous duplication facilitates rapid evolution and diversification of gene families. Mol Biol Evol. 29:2019–29. 22337864 10.1093/molbev/mss068

[bib46] Chippaux J-P, Williams V, White J. 1991. Snake venom variability: methods of study, results and interpretation. Toxicon. 29:1279–303. 1814005 10.1016/0041-0101(91)90116-9

[bib47] Christian K, Webb JK, Schultz T, Green B. 2007. Effects of seasonal variation in prey abundance on field metabolism, water flux, and activity of a tropical ambush foraging snake. Physiol Biochem Zool. 80:522–33. 17717815 10.1086/519959

[bib48] Cipriani V, Debono J, Goldenberg J, Jackson TNW, Arbuckle K, Dobson J, Koludarov I, Li B, Hay C, Dunstan N et al. 2017. Correlation between ontogenetic dietary shifts and venom variation in Australian brown snakes (*Pseudonaja*). Comp Biochem Physiol Part C: Toxicol Pharmacol. 197:53–60. 10.1016/j.cbpc.2017.04.007 28457945

[bib49] Colis-Torres A, Neri-Castro E, Strickland JL, Olvera-Rodríguez A, Borja M, Calvete J, Jones J, Parkinson CL, Bañuelos J, López De León J et al. 2022. Intraspecific venom variation of Mexican west coast rattlesnakes (*Crotalus basiliscus*) and its implications for antivenom production. Biochimie. 192:111–24. 34656669 10.1016/j.biochi.2021.10.006

[bib50] Coppinger GE, Stewart AJ, Borden JA, Strickland JL. 2025. *Thamnophis sirtalis* and their toxic relationship: testing for intraspecific venom variation in common garter snakes. Toxicon. 253:108185. 39615846 10.1016/j.toxicon.2024.108185

[bib51] Daltry JC, Wüster W, Thorpe RS. 1996. Diet and snake venom evolution. Nature. 379:537–40. 8596631 10.1038/379537a0

[bib52] Damm M, Avella I, Merzara R, Lucchini N, Buldain J, Corga F, Bouazza A, Fahd S, Süssmuth RD, Martínez-Freiría F. 2024. Venom variation among the three subspecies of the North African mountain viper *Vipera monticola* Saint Girons 1953. Biochimie. 227:152–60. 39029575 10.1016/j.biochi.2024.07.008

[bib53] Da Silva Aguiar W, Da Costa Galizio N, Sant’Anna SS, Silveira GPM, De Souza Rodrigues F, Grego KF, De Morais-Zani K, Tanaka-Azevedo AM. 2020. Ontogenetic study of *Bothrops jararacussu* venom composition reveals distinct profiles. Toxicon. 186:67–77. 32768441 10.1016/j.toxicon.2020.07.030

[bib54] Davies E-L, Arbuckle K. 2019. Coevolution of snake venom toxic activities and diet: evidence that ecological generalism favours toxicological diversity. Toxins. 11:711. 31817769 10.3390/toxins11120711 PMC6950196

[bib55] Davis Rabosky AR, Cox CL, Rabosky DL, Title PO, Holmes IA, Feldman A, McGuire JA. 2016. Coral snakes predict the evolution of mimicry across New World snakes. Nat Commun. 7:11484. 27146100 10.1038/ncomms11484 PMC4858746

[bib56] De SS . 1944. Physicochemical studies on hemolysin. J Indian Chem Soc. 21:290.

[bib57] Deka A, Bhatia S, Santra V, Bharti OK, Lalremsanga HT, Martin G, Wüster W, Owens JB, Graham S, Doley R et al. 2023. Multilevel comparison of Indian *Naja* venoms and their cross-reactivity with Indian polyvalent antivenoms. Toxins. 15:258. 37104196 10.3390/toxins15040258 PMC10142961

[bib58] De Wit CA . 1982. Resistance of the prairie vole (*Microtus ochrogaster*) and the woodrat (*Neotoma floridana*), in Kansas, to venom of the Osage copperhead (*Agkistrodon contortrix phaeogaster*). Toxicon. 20:709–14. 6753240 10.1016/0041-0101(82)90119-2

[bib59] Dhananjaya BL, D’Souza CJM. 2010. The pharmacological role of nucleotidases in snake venoms. Cell Biochem Funct. 28:171–7. 20186872 10.1002/cbf.1637

[bib60] Dobson J, Chowdhury A, Tai-A-Pin J, van der Ploeg H, Gillett A, Fry BG. 2024. The clot thickens: differential coagulotoxic and cardiotoxic activities of Anguimorpha lizard venoms. Toxins. 16:283. 38922177 10.3390/toxins16060283 PMC11209219

[bib61] Doley R, Mackessy SP, Kini RM. 2009. Role of accelerated segment switch in exons to alter targeting (ASSET) in the molecular evolution of snake venom proteins. BMC Evol Biol. 9:146. 19563684 10.1186/1471-2148-9-146 PMC2711939

[bib62] Drabeck DH, Dean AM, Jansa SA. 2015. Why the honey badger don’t care: convergent evolution of venom-targeted nicotinic acetylcholine receptors in mammals that survive venomous snake bites. Toxicon. 99:68–72. 25796346 10.1016/j.toxicon.2015.03.007

[bib63] Drukewitz SH, Von Reumont BM. 2019. The significance of comparative genomics in modern evolutionary venomics. Front Ecol Evol. 7:163.

[bib64] Du X-Y, Clemetson KJ. 2002. Snake venom l-amino acid oxidases. Toxicon. 40:659–65. 12175601 10.1016/s0041-0101(02)00102-2

[bib65] Esquerré D, Keogh JS, Dashevsky D, Bioleau J, Carroll A, Dunstan N, Mikheyev AS. 2026. Unlocking the venom vault: museum venomics reveals an untapped biochemical archive in natural history collections. bioRxiv. 10.64898/2026.05.22.727068:.

[bib66] Ferreira-Rodrigues SC, Silva RCC, Trevisan M, Rodrigues PSM, Del-Rei THM, Sousa LF, Vilarinho ARG, Lima CA, Rodrigues JL, Silva MMR et al. 2024. Ontogenetic and sexual differences in the venom of *Bothrops moojeni*: insights from a litter and its mother. Braz J Biol. 84:e279474. 38747862 10.1590/1519-6984.279474

[bib67] Fontana F . 1781. Traité sur le Vénin de la Vipère Florence.

[bib68] Fox JW, Serrano SMT. 2008. Insights into and speculations about snake venom metalloproteinase (SVMP) synthesis, folding and disulfide bond formation and their contribution to venom complexity. The FEBS J. 275:3016–30. 18479462 10.1111/j.1742-4658.2008.06466.x

[bib69] Frobert Y, Créminon C, Cousin X, Rémy M-H, Chatel J-M, Bon S, Bon C, Grassi J. 1997. Acetylcholinesterases from Elapidae snake venoms: biochemical, immunological and enzymatic characterization. Biochim Biophys Acta Protein Struct Mol Enzymol. 1339:253–67. 10.1016/s0167-4838(97)00009-5 9187246

[bib70] Fry BG, Roelants K, Winter K, Hodgson WC, Griesman L, Kwok HF, Scanlon D, Karas J, Shaw C, Wong L et al. 2010. Novel venom proteins produced by differential domain-expression strategies in beaded lizards and Gila monsters (genus *Heloderma*). Mol Biol Evol. 27:395–407. 19837656 10.1093/molbev/msp251

[bib71] Fry BG, Scheib H, Van Der Weerd L, Young B, McNaughtan J, Ramjan SFR, Vidal N, Poelmann RE, Norman JA. 2008. Evolution of an arsenal. Mol Cell Proteomics. 7:215–46. 17855442 10.1074/mcp.M700094-MCP200

[bib72] Fry BG, Vidal N, Norman JA, Vonk FJ, Scheib H, Ramjan SFR, Kuruppu S, Fung K, Blair Hedges S, Richardson MK et al. 2006. Early evolution of the venom system in lizards and snakes. Nature. 439:584–8. 16292255 10.1038/nature04328

[bib73] Fry BG, Wüster W, Kini RM, Brusic V, Khan A, Venkataraman D, Rooney AP. 2003. Molecular evolution and phylogeny of elapid snake venom three-finger toxins. J Mol Evol. 57:110–29. 12962311 10.1007/s00239-003-2461-2

[bib74] Furtado MFD, Travaglia-Cardoso SR, Rocha MMT. 2006. Sexual dimorphism in venom of *Bothrops jararaca* (Serpentes: Viperidae). Toxicon. 48:401–10. 16889808 10.1016/j.toxicon.2006.06.005

[bib75] Gibbs HL, Mackessy SP. 2009. Functional basis of a molecular adaptation: prey-specific toxic effects of venom from *Sistrurus* rattlesnakes. Toxicon. 53:672–9. 19673082 10.1016/j.toxicon.2009.01.034

[bib76] Gibbs HL, Rossiter W. 2008. Rapid evolution by positive selection and gene gain and loss: PLA2 venom genes in closely related *Sistrurus* rattlesnakes with divergent diets. J Mol Evol. 66:151–66. 18253686 10.1007/s00239-008-9067-7

[bib77] Gibbs HL, Sanz L, Pérez A, Ochoa A, Hassinger ATB, Holding ML, Calvete JJ. 2020. The molecular basis of venom resistance in a rattlesnake squirrel predator-prey system. Mol Ecol. 00:1–18. 10.1111/mec.15529 32593182

[bib78] Gibbs HL, Sanz L, Sovic MG, Calvete JJ. 2013. Phylogeny-based comparative analysis of venom proteome variation in a clade of rattlesnakes (*Sistrurus* sp.). PLoS ONE. 8:e67220. 23826238 10.1371/journal.pone.0067220 PMC3691181

[bib79] Girish KS, Jagadeesha DK, Rajeev KB, Kemparaju K. 2002. Snake venom hyaluronidase: an evidence for isoforms and extracellular matrix degradation. Mol Cell Biochem. 240:105–10. 12487377 10.1023/a:1020651607164

[bib80] Glenn JL, Straight R. 1977. The midget faded rattlesnake (*Crotalus viridis concolor*) venom: lethal toxicity and individual variability. Toxicon. 15:129–32. 854933 10.1016/0041-0101(77)90031-9

[bib81] Glenn JL, Straight R. 1978. Mojave rattlesnake *Crotalus scutulatus scutulatus* venom: variation in toxicity with geographical origin. Toxicon. 16:81–4. 622731 10.1016/0041-0101(78)90065-x

[bib82] Glenn JL, Straight RC. 1989. Intergradation of two different venom populations of the Mojave rattlesnake (*Crotalus scutulatus scutulatus*) in Arizona. Toxicon. 27:411–8. 2499081 10.1016/0041-0101(89)90203-1

[bib83] Glenn JL, Straight RC. 1990. Venom characteristics as an indicator of hybridization between *Crotalus viridis viridis* and *Crotalus scutulatus scutulatus* in New Mexico. Toxicon. 28:857–62. 2120798 10.1016/s0041-0101(09)80008-1

[bib84] Glenn JL, Straight RC, Wolfe MC, Hardy DL. 1983. Geographical variation in *Crotalus scutulatus scutulatus* (Mojave rattlesnake) venom properties. Toxicon. 21:119–30. 6342208 10.1016/0041-0101(83)90055-7

[bib85] Goldenberg J, Cipriani V, Jackson TNW, Arbuckle K, Debono J, Dashevsky D, Panagides N, Ikonomopoulou MP, Koludarov I, Li B et al. 2018. Proteomic and functional variation within black snake venoms (Elapidae: *Pseudechis*). Comp Biochem Physiol Part C Toxicol Pharmacol. 205:53–61. 10.1016/j.cbpc.2018.01.001 29353015

[bib86] Gómez A, Segura Á, Solano G, Chacón D, Corrales G. 2021. Influence of sexual dimorphism on venom composition in *Bothrops asper* and *Crotalus simus* (Serpentes: Viperidae) and its potential implications on the snake antivenom production. Toxicon. 204:1–4. 34687716 10.1016/j.toxicon.2021.10.008

[bib87] Gregory-Dwyer VM, Egen NB, Bosisio AB, Righetti PG, Russell FE. 1986. An isoelectric focusing study of seasonal variation in rattlesnake venom proteins. Toxicon. 24:995–1000. 3824406 10.1016/0041-0101(86)90005-x

[bib88] Gubenšek F, Sket D, Turk V, Lebez D. 1974. Fractionation of *Vipera ammodytes* venom and seasonal variation of its composition. Toxicon. 12:167–71. 4852085 10.1016/0041-0101(74)90241-4

[bib89] Guércio RA, Shevchenko A, Shevchenko A, López-Lozano JL, Paba J, Sousa MV, Ricart CA. 2006. Ontogenetic variations in the venom proteome of the Amazonian snake *Bothrops atrox*. Proteome Sci. 4:11. 16689994 10.1186/1477-5956-4-11 PMC1483819

[bib90] Guo C, Liu S, Yao Y, Zhang Q, Sun M-Z. 2012. Past decade study of snake venom l-amino acid oxidase. Toxicon. 60:302–11. 22579637 10.1016/j.toxicon.2012.05.001

[bib91] Gutiérrez J . 2000. Snake venom metalloproteinases: their role in the pathogenesis of local tissue damage. Biochimie. 82:841–50. 11086214 10.1016/s0300-9084(00)01163-9

[bib92] Gutiérrez JM, Lomonte B, León G, Alape-Girón A, Flores-Díaz M, Sanz L, Angulo Y, Calvete JJ. 2009. Snake venomics and antivenomics: proteomic tools in the design and control of antivenoms for the treatment of snakebite envenoming. J Proteomics. 72:165–82. 19344652 10.1016/j.jprot.2009.01.008

[bib93] Hargreaves AD, Swain MT, Logan DW, Mulley JF. 2014. Testing the toxicofera: comparative transcriptomics casts doubt on the single, early evolution of the reptile venom system. Toxicon. 92:140–56. 25449103 10.1016/j.toxicon.2014.10.004

[bib94] Harrison CM, Colbert J, Richter CJ, McDonald PJ, Trumbull LM, Ellsworth SA, Hogan MP, Rokyta DR, Margres MJ. 2022. Using morphological, genetic, and venom analyses to present current and historic evidence of *Crotalus horridus* x *adamanteus* hybridization on Jekyll Island, Georgia. Southeastern Naturalist. 21:158–74.

[bib95] Healy K, Carbone C, Jackson AL. 2019. Snake venom potency and yield are associated with prey-evolution, predator metabolism and habitat structure. Ecol Lett. 22:527–37. 30616302 10.1111/ele.13216

[bib96] Heatwole H, Poran N, King P. 1999. Ontogenetic changes in the resistance of bullfrogs (*Rana catesbeiana*) to the venom of copperheads (*Agkistrodon contortrix contortrix*) and cottonmouths (*Agkistrodon piscivorus piscivorus*). Copeia. 1999:808–14.

[bib97] Heatwole H, Powell J. 1998. Resistance of eels (*Gymnothorax*) to the venom of sea kraits (*Laticauda colubrina*): a test of coevolution. Toxicon. 36:619–25. 9643474 10.1016/s0041-0101(97)00081-0

[bib98] Heptinstall TC, Rosales García RA, Rautsaw RM, Myers EA, Holding ML, Mason AJ, Hofmann EP, Schramer TD, Hogan MP, Borja M et al. 2025. Dietary breadth predicts toxin expression complexity in the venoms of North American gartersnakes. Integr Organ Biol. 7::obaf003 10.1093/iob/obaf003 PMC11822205 39959576

[bib99] Heyborne WH, Mackessy SP. 2021. Venoms of new world vinesnakes (*Oxybelis aeneus* and *O. fulgidus*). Toxicon. 190:22–30. 33307109 10.1016/j.toxicon.2020.12.002

[bib100] Holding ML, Biardi JE, Gibbs HL. 2016a. Coevolution of venom function and venom resistance in a rattlesnake predator and its squirrel prey. Proc R Soc B, 283:20152841. 10.1098/rspb.2015.2841 PMC4855376 27122552

[bib101] Holding ML, Drabeck DH, Jansa SA, Gibbs HL. 2016b. Venom resistance as a model for understanding the molecular basis of complex coevolutionary adaptations. Integr Comp Biol. 56:1032–43. 27444525 10.1093/icb/icw082

[bib102] Holding ML, Margres MJ, Rokyta DR, Gibbs HL. 2018. Local prey community composition and genetic distance predict venom divergence among populations of the northern Pacific rattlesnake (*Crotalus oreganus*). J Evol Biol. 31:1513–28. 29959877 10.1111/jeb.13347

[bib103] Holding ML, Strickland JL, Rautsaw RM, Hofmann EP, Mason AJ, Hogan MP, Nystrom GS, Ellsworth SA, Colston TJ, Borja M et al. 2021. Phylogenetically diverse diets favor more complex venoms in North American pitvipers. Proc Natl Acad Sci USA. 118:e2015579118. 33875585 10.1073/pnas.2015579118 PMC8092465

[bib104] Huang T-F, Liu CZ, Yang S-H. 1995. Aggretin, a novel platelet-aggregation inducer from snake (*Calloselasma rhodostoma*) venom, activates phospholipase C by acting as a glycoprotein Ia/IIa agonist. Biochem J. 309:1021–27. 7639679 10.1042/bj3091021 PMC1135733

[bib105] Hus KK, Buczkowicz J, Pietrowska M, Petrilla V, Petrillová M, Legáth J, Litschka-Koen T, Bocian A. 2024. Venom diversity in *Naja mossambica*: insights from proteomic and immunochemical analyses reveal intraspecific differences. PLoS Negl Trop Dis, 18:e0012057. 38557658 10.1371/journal.pntd.0012057 PMC11008852

[bib106] Izidoro LFM, Sobrinho JC, Mendes MM, Costa TR, Grabner AN, Rodrigues VM, Da Silva SL, Zanchi FB, Zuliani JP, Fernandes CFC, Calderon LA, Stábeli RG, Soares AM. 2014. Snake venom L-amino acid oxidases: trends in pharmacology and biochemistry. BioMed Res Int. 2014:1–19. 10.1155/2014/196754 PMC3971498 24738050

[bib107] Jackson T, Koludarov I, Ali S, Dobson J, Zdenek C, Dashevsky D, Op D, Brouw B, Masci P, Nouwens A et al. 2016. Rapid radiations and the race to redundancy: an investigation of the evolution of Australian elapid snake venoms. Toxins. 8:309. 27792190 10.3390/toxins8110309 PMC5127106

[bib108] Jansa SA, Voss RS. 2011. Adaptive evolution of the venom-targeted vWF protein in opossums that eat pitvipers. PLoS One. 6:e20997. 21731638 10.1371/journal.pone.0020997 PMC3120824

[bib109] Jímenez-Porras JM . 1964. Intraspecific variations in composition of venom of the jumping viper, *Bothrops nummifera*. Toxicon. 2:187–95. 14298226 10.1016/0041-0101(64)90021-2

[bib110] Jorge da Silva N, Aird SD. 2001. Prey specificity, comparative lethality and compositional differences of coral snake venoms. Comp Biochem Physiol Part C Toxicol Pharmacol. 128:425–56. 10.1016/s1532-0456(00)00215-5 11255115

[bib111] Juarez P, Comas I, Gonzalez-Candelas F, Calvete JJ. 2008. Evolution of snake venom disintegrins by positive Darwinian selection. Mol Biol Evol. 25:2391–407. 18701431 10.1093/molbev/msn179

[bib112] Juárez P, Sanz L, Calvete JJ. 2004. Snake venomics: characterization of protein families in *Sistrurus barbouri* venom by cysteine mapping, *N*-terminal sequencing, and tandem mass spectrometry analysis. Proteomics. 4:327–38. 14760702 10.1002/pmic.200300628

[bib113] Junqueira-de-Azevedo I, Campos P, Ching A, Mackessy S. 2016. Colubrid venom composition: an -omics perspective. Toxins. 8:230. 27455326 10.3390/toxins8080230 PMC4999846

[bib114] Junqueira-de-Azevedo ILM, Ching ATC, Carvalho E, Faria F, Nishiyama MY, Ho PL, Diniz MRV. 2006. *Lachesis muta* (Viperidae) cDNAs reveal diverging pit viper molecules and scaffolds typical of Cobra (Elapidae) venoms: implications for snake toxin repertoire evolution. Genetics. 173:877–89. 16582429 10.1534/genetics.106.056515 PMC1526512

[bib115] Kazandjian TD, Petras D, Robinson SD, van Thiel J, Greene HW, Arbuckle K, Barlow A, Carter DA, Wouters RM, Whiteley G, Wagstaff SC, Arias A, 2021. Convergent evolution of pain-inducing defensive venom components in spitting cobras. Science. 371:386–90. 33479150 10.1126/science.abb9303 PMC7610493

[bib116] Kerkis I, Hayashi MAF, Prieto Da Silva ARB, Pereira A, De Sá Júnior PL, Zaharenko AJ, Rádis-Baptista G, Kerkis A, Yamane T. 2014. State of the art in the studies on crotamine, a cell penetrating peptide from South American rattlesnake. BioMed Res Int. 2014:1–9. 10.1155/2014/675985 PMC3914522 24551848

[bib117] Kilmon JA . 1976. High tolerance to snake venom by the Virginia opossum, *Didelphis virginiana*. Toxicon. 14:337–40. 960117 10.1016/0041-0101(76)90032-5

[bib118] Kini RM . 2003. Excitement ahead: structure, function and mechanism of snake venom phospholipase A2 enzymes. Toxicon. 42:827–40. 15019485 10.1016/j.toxicon.2003.11.002

[bib119] Kini RM . 2005. Serine proteases affecting blood coagulation and fibrinolysis from snake venoms. Pathophysiol Haemos Thromb. 34:200–4. 10.1159/000092424 16707928

[bib120] Kock MA, Hew BE, Bammert H, Fritzinger DC, Vogel C-W. 2004. Structure and function of recombinant cobra venom factor. J Biol Chem. 279:30836–43. 15131128 10.1074/jbc.M403196200

[bib121] Koludarov I, Jackson T, Sunagar K, Nouwens A, Hendrikx I, Fry B. 2014. Fossilized venom: the unusually conserved venom profiles of *Heloderma* species (beaded lizards and Gila monsters). Toxins. 6:3582–95. 25533521 10.3390/toxins6123582 PMC4280549

[bib122] Kordiš D, Gubenšek F. 2000. Adaptive evolution of animal toxin multigene families. Gene. 261:43–52. 11164036 10.1016/s0378-1119(00)00490-x

[bib123] Kostiza T, Meier J. 1996. Nerve growth factors from snake venoms: chemical properties, mode of action and biological significance. Toxicon. 34:787–806. 8843580 10.1016/0041-0101(96)00023-2

[bib124] Koua D, Ebou A, Habbouche Z, Ballouard J-M, Caron S, Bonnet X, Dutertre S. 2022. Proteomic insight into the venom composition of the largest European rear-fanged snake, *Malpolon monspessulanus monspessulanus*. Toxicon: X: X 15:100130. 35721600 10.1016/j.toxcx.2022.100130 PMC9201006

[bib125] Kunalan S, Othman I, Syed Hassan S, Hodgson WC. 2018. Proteomic characterization of two medically important Malaysian snake venoms, *Calloselasma rhodostoma* (Malayan pit viper) and *Ophiophagus hannah* (king cobra). Toxins. 10:434. 30373186 10.3390/toxins10110434 PMC6266455

[bib126] Lakušić M, Damm M, Bjelica V, Anđelković M, Tomović L, Bonnet X, Arsovski D, Süssmuth RD, Calvete JJ, Martínez-Freiría F. 2025. Ontogeny, not prey availability, underlies allopatric venom variability in insular and mainland populations of *Vipera ammodytes*. J Proteomics. 310:105320. 39306033 10.1016/j.jprot.2024.105320

[bib127] Lakušić M, Martínez-Freiría F, Anđelković M, Hempel B-F. 2025. Beyond sexual maturity: importance of dietary changes in venom variation in *Vipera ammodytes*. Toxicon. 257:108291. 39983996 10.1016/j.toxicon.2025.108291

[bib128] Li M, Fry BG, Kini RM. 2005. Putting the brakes on snake venom evolution: the unique molecular evolutionary patterns of *Aipysurus eydouxii* (marbled eea snake) phospholipase A2 toxins. Mol Biol Evol. 22:934–41. 15635056 10.1093/molbev/msi077

[bib129] Lüddecke T, Avella I, Damm M, Schulte L, Eichberg J, Hardes K, Schiffmann S, Henke M, Timm T, Lochnit G et al. 2024. The toxin diversity, cytotoxicity, and enzymatic activity of Cape cobra (*Naja nivea*) Venom. Toxins. 16:438. 39453214 10.3390/toxins16100438 PMC11511112

[bib130] Lüddecke T, Paas A, Harris RJ, Talmann L, Kirchhoff KN, Billion A, Hardes K, Steinbrink A, Gerlach D, Fry BG et al. 2023. Venom biotechnology: casting light on nature’s deadliest weapons using synthetic biology. Front Bioeng Biotechnol. 11:1166601. 37207126 10.3389/fbioe.2023.1166601 PMC10188951

[bib131] Lyons K, Dugon MM, Healy K. 2020. Diet breadth mediates the prey specificity of venom potency in snakes. Toxins. 12:74. 31979380 10.3390/toxins12020074 PMC7076792

[bib132] Machado Braga JR, De Morais-Zani K, Pereira DDS, Sant’Anna SS, Da Costa Galizio N, Tanaka-Azevedo AM, Gomes Vilarinho AR, Rodrigues JL, Teixeira Da Rocha MM, 2020.Sexual and ontogenetic variation of *Bothrops leucurus* venom. Toxicon. 184:127–35. 32553734 10.1016/j.toxicon.2020.05.028

[bib133] Macías-Rodríguez EF, Díaz-Cárdenas CO, Gatica-Colima AB, Plenge-Tellechea LF. 2014. Seasonal variation in protein content and PLA2 activity of *Crotalus molossus molossus* venom from captive and wild specimens. AU. 24:38–47.

[bib134] Mackessy SP . 1988. Venom ontogeny in the Pacific rattlesnakes *Crotalus viridis helleri* and *C. v. oreganus*. Copeia. 1988:92–101.

[bib135] Mackessy SP . 2002. Biochemistry and pharmacology of colubrid snake venoms. J Toxicol Toxin Rev. 21:43–83.

[bib136] Mackessy SP . 2008. Venom composition in rattlesnakes: trends and biological significance. In: The biology of rattlesnakes. Loma Linda (CA): Loma Linda University Press. p.495–510.

[bib137] Mackessy SP (Ed.). 2010a. Handbook of venoms and toxins of reptiles. Boca Raton (FL): CRC Press.

[bib138] Mackessy SP . 2010b. Evolutionary trends in venom composition in the western rattlesnakes (*Crotalus viridis* sensu lato): toxicity vs. tenderizers. Toxicon. 55:1463–74. 20227433 10.1016/j.toxicon.2010.02.028

[bib139] Mackessy SP . 2021. Handbook of venoms and toxins of reptiles. 2nd ed. Boca Raton (FL): CRC press.

[bib140] Mackessy SP . 2022. Venom production and secretion in reptiles. J Exp Biol. 225:jeb227348. 35363854 10.1242/jeb.227348

[bib141] Mackessy SP, Leroy J, Mociño-Deloya E, Setser K, Bryson RW, Saviola AJ. 2018. Venom ontogeny in the Mexican lance-headed rattlesnake (*Crotalus polystictus*). Toxins. 10:271. 29970805 10.3390/toxins10070271 PMC6070973

[bib142] Mackessy SP, Sixberry NM, Heyborne WH, Fritts T. 2006. Venom of the brown treesnake, *Boiga irregularis*: ontogenetic shifts and taxa-specific toxicity. Toxicon. 47:537–48. 16545413 10.1016/j.toxicon.2006.01.007

[bib143] Mackessy SP, Tu AT. 1993. Biology of the sea snakes and biochemistry of their venoms. In: Toxin-related diseases: poisons originating from plants, animals and spoilage. New Delhi: Oxford & IBH Publishing Co. p.543.

[bib144] Mackessy SP, Williams K, Ashton KG. 2003. Ontogenetic variation in venom composition and diet of *Crotalus oreganus concolor*: a case of venom paedomorphosis?. Copeia. 2003:769–82.

[bib145] Margres MJ, Wray KP, Sanader D, McDonald PJ, Trumbull LM, Patton AH, Rokyta DR. 2021. Varying intensities of introgression obscure incipient venom-associated speciation in the timber rattlesnake (*Crotalus horridus*). Toxins. 13:782. 34822565 10.3390/toxins13110782 PMC8625053

[bib146] Matsui T, Fujimura Y, Titani K. 2000. Snake venom proteases affecting hemostasis and thrombosis. Biochim Biophys Acta Protein Struct Mol Enzymol. 1477:146–56. 10.1016/s0167-4838(99)00268-x 10708855

[bib147] Mehr S, Castoe T, Daly M, Jungo F, Kirchhoff KN, Koludarov I, Mackessy SP, Macrander J, Naidu P, Modica MV et al. 2026. A proposed unified, scalable platform for integrative research on venomous species. GigaScience. 184: giaf153 10.1093/gigascience/giaf153 41618863

[bib148] Menezes MC, Furtado MF, Travaglia-Cardoso SR, Camargo ACM, Serrano SMT. 2006. Sex-based individual variation of snake venom proteome among eighteen *Bothrops jararaca* siblings. Toxicon. 47:304–12. 16373076 10.1016/j.toxicon.2005.11.007

[bib149] Minton SA, Weinstein SA. 1986. Geographic and ontogenic variation in venom of the western diamondback rattlesnake (*Crotalus atrox*). Toxicon. 24:71–80. 3513378 10.1016/0041-0101(86)90167-4

[bib150] Modahl CM, Han SX, Van Thiel J, Vaz C, Dunstan NL, Frietze S, Jackson TNW, Mackessy SP, Kini RM. 2024. Distinct regulatory networks control toxin gene expression in elapid and viperid snakes. BMC Genomics. 25:186. 38365592 10.1186/s12864-024-10090-y PMC10874052

[bib151] Modahl CM, Mackessy SP. 2019. Venoms of rear-fanged snakes: new proteins and novel activities. Front Ecol Evol. 7:279.

[bib152] Modahl CM, Mrinalini FS, Mackessy SP. 2018. Adaptive evolution of distinct prey-specific toxin genes in rear-fanged snake venom. Proc R Soc B. 285:20181003. 10.1098/rspb.2018.1003 PMC6111164 30068680

[bib153] Modahl CM, Mukherjee AK, Mackessy SP. 2016. An analysis of venom ontogeny and prey-specific toxicity in the monocled cobra (*Naja kaouthia*). Toxicon. 119:8–20. 27163885 10.1016/j.toxicon.2016.04.049

[bib154] Montecucco C, Gutiérrez JM, Lomonte B. 2008. Cellular pathology induced by snake venom phospholipase A2 myotoxins and neurotoxins: common aspects of their mechanisms of action. Cell Mol Life Sci. 65:2897–912. 18563294 10.1007/s00018-008-8113-3 PMC11131735

[bib155] Mora-Obando D, Salazar-Valenzuela D, Pla D, Lomonte B, Guerrero-Vargas JA, Ayerbe S, Gibbs HL, Calvete JJ. 2020. Venom variation in *Bothrops asper* lineages from North-Western South America. J Proteomics. 229:103945. 32829066 10.1016/j.jprot.2020.103945

[bib156] Morita T . 2005. Structures and functions of snake venom CLPs (C-type lectin-like proteins) with anticoagulant-, procoagulant-, and platelet-modulating activities. Toxicon. 45:1099–114. 15922777 10.1016/j.toxicon.2005.02.021

[bib157] Mukherjee AK, Mackessy SP, Dutta S. 2014. Characterization of a Kunitz-type protease inhibitor peptide (Rusvikunin) purified from *Daboia russelii russelii* venom. Int J Biol Macromol. 67:154–62. 24632346 10.1016/j.ijbiomac.2014.02.058

[bib158] Muschick M, Indermaur A, Salzburger W. 2012. Convergent evolution within an adaptive radiation of cichlid fishes. Current Biol. 22:2362–8. 10.1016/j.cub.2012.10.048 23159601

[bib159] Myers EA, Rautsaw RM, Borja M, Jones J, Grünwald CI, Holding ML, Grazziotin FG, Parkinson CL. 2024. Phylogenomic discordance is driven by wide-spread introgression and incomplete lineage sorting during rapid species diversification within rattlesnakes (Viperidae: *Crotalus* and *Sistrurus*). Systematic Biol. 73:722–41. 10.1093/sysbio/syae018 PMC11906154 38695290

[bib160] Nair DG, Fry BG, Alewood P, Kumar PP, Kini RM . 2007. Antimicrobial activity of omwaprin, a new member of the waprin family of snake venom proteins. Biochem J. 402:93–104. 17044815 10.1042/BJ20060318 PMC1783991

[bib161] Nei M, Gu X, Sitnikova T. 1997. Evolution by the birth-and-death process in multigene families of the vertebrate immune system. Proc Natl Acad Sci USA. 94:7799–806. 9223266 10.1073/pnas.94.15.7799 PMC33709

[bib162] Neri-Castro E, Strickland JL, Carbajal-Márquez RA, Zuñiga Adán J, Ponce-López R, Olvera-Rodríguez F, Alagón A. 2022. Characterization of the venom and external morphology of a natural hybrid between *Crotalus atrox* and *Crotalus mictlantecuhtli*. Toxicon. 207:43–7. 35007607 10.1016/j.toxicon.2022.01.003

[bib163] Nie X, Chen Q, Wang C, Huang W, Lai R, Lu Q, He Q, Yu X. 2022. Venom variation of neonate and adult Chinese cobras in captivity concerning their foraging strategies. Toxins. 14:598. 36136536 10.3390/toxins14090598 PMC9501182

[bib164] Ogawa T, Chijiwa T, Oda-Ueda N, Ohno M. 2005. Molecular diversity and accelerated evolution of C-type lectin-like proteins from snake venom. Toxicon. 45:1–14. 15581677 10.1016/j.toxicon.2004.07.028

[bib165] Ogawa T, Oda-Ueda N, Hisata K, Nakamura H, Chijiwa T, Hattori S, Isomoto A, Yugeta H, Yamasaki S, Fukumaki Y et al. 2019. Alternative mRNA splicing in three venom families underlying a possible production of divergent venom proteins of the Habu snake, *Protobothrops flavoviridis*. Toxins. 11:581. 31600994 10.3390/toxins11100581 PMC6832531

[bib166] Oguiura N, Boni-Mitake M, Rádis-Baptista G. 2005. New view on crotamine, a small basic polypeptide myotoxin from South American rattlesnake venom. Toxicon. 46:363–70. 16115660 10.1016/j.toxicon.2005.06.009

[bib167] Oh AMF, Tan CH, Tan KY, Quraishi NH, Tan NH. 2019. Venom proteome of *Bungarus sindanus* (Sind krait) from Pakistan and in vivo cross-neutralization of toxicity using an Indian polyvalent antivenom. J Proteomics. 193:243–54. 30385415 10.1016/j.jprot.2018.10.016

[bib168] Ohno M, Chijiwa T, Oda-Ueda N, Ogawa T, Hattori S. 2003. Molecular evolution of myotoxic phospholipases A2 from snake venom. Toxicon. 42:841–54. 15019486 10.1016/j.toxicon.2003.11.003

[bib169] Ohno S . 1970. Evolution by gene duplication. 1st ed. Heidelberg: Springer Berlin.

[bib170] Oliveira AL, Viegas MF, Da Silva SL, Soares AM, Ramos MJ, Fernandes PA. 2022. The chemistry of snake venom and its medicinal potential. Nat Rev Chem. 6:451–69. 35702592 10.1038/s41570-022-00393-7 PMC9185726

[bib171] OmPraba G, Chapeaurouge A, Doley R, Devi KR, Padmanaban P, Venkatraman C, Velmurugan D, Lin Q, Kini RM. 2010. Identification of a novel family of snake venom proteins Veficolins from *Cerberus rynchops* using a venom gland transcriptomics and proteomics approach. J Proteome Res. 9:1882–93. 20158271 10.1021/pr901044x

[bib172] Pahari S, Mackessy SP, Kini RM. 2007. The venom gland transcriptome of the desert massasauga rattlesnake (*Sistrurus catenatus edwardsii*): towards an understanding of venom composition among advanced snakes (Superfamily Colubroidea). BMC Mol Biol. 8:115. 18096037 10.1186/1471-2199-8-115 PMC2242803

[bib173] Pawlak J, Mackessy SP, Sixberry NM, Stura EA, Le Du MH, Ménez R, Foo CS, Ménez A, Nirthanan S, Kini RM. 2009. Irditoxin, a novel covalently linked heterodimeric three-finger toxin with high taxon-specific neurotoxicity. FASEB J. 23:534–45. 18952712 10.1096/fj.08-113555

[bib174] Perez JC, Haws WC, Garcia VE, Jennings BM. 1978a. Resistance of warm-blooded animals to snake venoms. Toxicon. 16:375–83. 684768 10.1016/0041-0101(78)90158-7

[bib175] Perez JC, Haws WC, Hatch CH. 1978b. Resistance of woodrats (*Neotoma micropus*) to *Crotalus atrox* venom. Toxicon. 16:198–200. 635934 10.1016/0041-0101(78)90039-9

[bib176] Perez JC, Pichyangkul S, Garcia VE. 1979. The resistance of three species of warm-blooded animals to western diamondback rattlesnake (*Crotalus atrox*) venom. Toxicon. 17:601–7. 524385 10.1016/0041-0101(79)90234-4

[bib177] Phillips MA, Waterman JM, Du Plessis P, Smit M, Bennett NC. 2012. No evidence for proteolytic venom resistance in southern African ground squirrels. Toxicon. 60:760–3. 22728461 10.1016/j.toxicon.2012.06.004

[bib178] Pimenta DC, Prezoto BC, Konno K, Melo RL, Furtado MF, Camargo ACM, Serrano SMT. 2007. Mass spectrometric analysis of the individual variability of *Bothrops jararaca* venom peptide fraction. Evidence for sex-based variation among the bradykinin-potentiating peptides. Rapid Comm Mass Spectrom. 21:1034–42. 10.1002/rcm.2931 17315274

[bib179] Pomento AM, Perry BW, Denton RD, Gibbs HL, Holding ML. 2016. No safety in the trees: local and species-level adaptation of an arboreal squirrel to the venom of sympatric rattlesnakes. Toxicon. 118:149–55. 27158112 10.1016/j.toxicon.2016.05.003

[bib180] Poran NS, Coss RG, Benjamini E. 1987. Resistance of California ground squirrels (*Spermophilus beecheyi*) to the venom of the northern Pacific rattlesnake (*Crotalus viridis oreganus*): a study of adaptive variation. Toxicon. 25:767–77. 3672545 10.1016/0041-0101(87)90127-9

[bib181] Pung YF, Wong PTH, Kumar PP, Hodgson WC, Kini RM . 2005. Ohanin, a novel protein from king cobra venom, induces hypolocomotion and hyperalgesia in mice. J Biol Chem. 13:13137–47. 10.1074/jbc.M414137200 15668253

[bib182] Qiao Z, Jones L, Bourke LA, Seneci L, Chowdhury A, Violette A, Fourmy R, Soria R, Aldridge M, Fry BG. 2024. Tiny but mighty: *Vipera ammodytes meridionalis* (eastern long-nosed viper) ontogenetic venom variations in procoagulant potency and the impact on antivenom efficacies. Toxins. 16:396. 39330854 10.3390/toxins16090396 PMC11436208

[bib183] Quijada-Mascareñas A, Wüster W. 2009. Recent advances in venomous snake systematics. In: Handbook of Venoms and Toxins of Reptiles, Boca Raton (FL): CRC Press. p.23–62.

[bib184] Rashmi U, Khochare S, Attarde S, Laxme RRS, Suranse V, Martin G, Sunagar K. 2021. Remarkable intrapopulation venom variability in the monocellate cobra (*Naja kaouthia*) unveils neglected aspects of India’s snakebite problem. J Proteomics. 242:104256. 33957314 10.1016/j.jprot.2021.104256

[bib185] Redi F . 1664. Osservazioni intorno alle vipere Firenze: All’Insegna della Stella.

[bib186] Richards DP, Barlow A, Wüster W. 2012. Venom lethality and diet: differential responses of natural prey and model organisms to the venom of the saw-scaled vipers (*Echis*). Toxicon. 59:110–6. 22079297 10.1016/j.toxicon.2011.10.015

[bib187] Richards R, St Pierre L, Trabi M, Johnson LA, De Jersey J, Masci PP, Lavin MF. 2011. Cloning and characterisation of novel cystatins from elapid snake venom glands. Biochimie. 93:659–68. 21172403 10.1016/j.biochi.2010.12.008

[bib188] Robinson KE, Holding ML, Whitford MD, Saviola AJ, Yates JR, Clark RW. 2021. Phenotypic and functional variation in venom and venom resistance of two sympatric rattlesnakes and their prey. J Evol Biol. 34:1447–65. 34322920 10.1111/jeb.13907

[bib189] Rokyta DR, Margres MJ, Calvin K. 2015. Post-transcriptional mechanisms contribute little to phenotypic variation in snake venoms. G3 Genes|Genomes|Genetics. 5:2375–82. 26358130 10.1534/g3.115.020578 PMC4632057

[bib190] Rokyta DR, Wray KP, Lemmon AR, Lemmon EM, Caudle SB. 2011. A high-throughput venom-gland transcriptome for the eastern diamondback rattlesnake (*Crotalus adamanteus*) and evidence for pervasive positive selection across toxin classes. Toxicon. 57:657–71. 21255598 10.1016/j.toxicon.2011.01.008

[bib191] Roldán-Padrón O, Cruz-Pérez MS, Castro-Guillén JL, García-Arredondo JA, Mendiola-Olaya E, Saldaña-Gutiérrez C, Herrera-Paniagua P, Blanco-Labra A, García-Gasca T, 2022. Hybridization between *Crotalus aquilus* and *Crotalus polystictus* species: a comparison of their venom toxicity and enzymatic activities. Biology. 11:661. 35625389 10.3390/biology11050661 PMC9138290

[bib192] Rundus AS, Owings DH, Joshi SS, Chinn E, Giannini N. 2007. Ground squirrels use an infrared signal to deter rattlesnake predation. Proc Natl Acad Sci USA. 104:14372–6. 17704254 10.1073/pnas.0702599104 PMC1950100

[bib193] Russell FE, Bogert CM. 1981. Gila monster: its biology, venom and bite—A review. Toxicon. 19:341–59. 7018022 10.1016/0041-0101(81)90040-4

[bib194] Sánchez MN, Teibler GP, López CA, Mackessy SP, Peichoto ME. 2018. Assessment of the potential toxicological hazard of the green parrot snake (*Leptophis ahaetulla marginatus*): characterization of its venom and venom-delivery system. Toxicon. 148:202–12. 29705149 10.1016/j.toxicon.2018.04.027

[bib195] Sanggaard KW, Dyrlund TF, Thomsen LR, Nielsen TA, Brøndum L, Wang T, Thøgersen IB, Enghild JJ. 2015. Characterization of the Gila monster (*Heloderma suspectum suspectum*) venom proteome. J Proteomics. 117:1–11. 25603280 10.1016/j.jprot.2015.01.004

[bib196] Sanz L, Gibbs HL, Mackessy SP, Calvete JJ. 2006. Venom proteomes of closely related *Sistrurus* rattlesnakes with divergent diets. J Proteome Res. 5:2098–112. 16944921 10.1021/pr0602500

[bib197] Sanz L, Pla D, Pérez A, Rodríguez Y, Zavaleta A, Salas M, Lomonte B, Calvete J. 2016. Venomic analysis of the poorly studied desert coral snake, *Micrurus tschudii tschudii*, supports the 3FTx/PLA2 dichotomy across Micrurus venoms. Toxins. 8:178. 27338473 10.3390/toxins8060178 PMC4926144

[bib198] Sanz L, Quesada-Bernat S, Ramos T, Casais-e-Silva LL, Corrêa-Netto C, Silva-Haad JJ, Sasa M, Lomonte B, Calvete JJ. 2019. New insights into the phylogeographic distribution of the 3FTx/PLA2 venom dichotomy across genus *Micrurus* in South America. J Proteomics. 200:90–101. 30946991 10.1016/j.jprot.2019.03.014

[bib199] Sarangi N, Senji Laxme RR, Sunagar K. 2025. Significant serpents: predictive modelling of bioclimatic venom variation in Russell’s viper. PLoS Negl Trop Dis. 19:e0012949. 40208847 10.1371/journal.pntd.0012949 PMC11984747

[bib200] Saviola AJ, Pla D, Sanz L, Castoe TA, Calvete JJ, Mackessy SP. 2015. Comparative venomics of the Prairie Rattlesnake (*Crotalus viridis viridis*) from Colorado: identification of a novel pattern of ontogenetic changes in venom composition and assessment of the immunoreactivity of the commercial antivenom CroFab®. J Proteomics. 121:28–43. 25819372 10.1016/j.jprot.2015.03.015

[bib201] Schaeffer R, Pascolutti VJ, Jackson TNW, Arbuckle K. 2023. Diversity begets diversity when diet drives snake venom evolution, but evenness rather than richness is what counts. Toxins. 15:251. 37104189 10.3390/toxins15040251 PMC10142186

[bib202] Schulte L, Engelhardt M, Eichberg J, Cabrera-Orefice A, Wölfel H, Damm M, Avella I, Kreuels B, Hardes K, Eble JA et al. 2026a. Comparison of proteomes and biofunctional properties of male and female common adder (*Vipera berus*) venoms. Biochimie. 244:40–53. 41643806 10.1016/j.biochi.2026.01.018

[bib203] Schulte L, Engelhardt M, Eichberg J, Cabrera-Orefice A, Wölfel H, Damm M, Avella I, Kreuels B, Hardes K, Eble JA et al. 2026b. Ontogenetic variation in composition and bioactivity of common adder (*Vipera berus*) venom revealed by genome-guided proteomics and in vitro functional assays. Biorxiv 10.64898/2026.02.09.704791.

[bib204] Schweitz H, Vigne P, Moinier D, Frelin C, Lazdunski M. 1992. A new member of the natriuretic peptide family is present in the venom of the green mamba (*Dendroaspis angusticeps*). J Biol Chem. 267:13928–32. 1352773

[bib205] Sciani JM, Pimenta DC. 2017. The modular nature of bradykinin-potentiating peptides isolated from snake venoms. J Venom Anim Toxins Incl Trop Dis. 23:45. 29090005 10.1186/s40409-017-0134-7 PMC5657115

[bib206] Senji Laxme RR, Khochare S, Bhatia S, Martin G, Sunagar K. 2024. From birth to bite: the evolutionary ecology of India’s medically most important snake venoms. BMC Biol. 22:161. 39075553 10.1186/s12915-024-01960-8 PMC11287890

[bib207] Shibata H, Chijiwa T, Oda-Ueda N, Nakamura H, Yamaguchi K, Hattori S, Matsubara K, Matsuda Y, Yamashita A, Isomoto A et al. 2018. The habu genome reveals accelerated evolution of venom protein genes. Sci Rep. 8:11300. 30050104 10.1038/s41598-018-28749-4 PMC6062510

[bib208] Sintiprungrat K, Watcharatanyatip K, Senevirathne WDST, Chaisuriya P, Chokchaichamnankit D, Srisomsap C, Ratanabanangkoon K. 2016. A comparative study of venomics of *Naja naja* from India and Sri Lanka, clinical manifestations and antivenomics of an Indian polyspecific antivenom. J Proteomics. 132:131–43. 26506536 10.1016/j.jprot.2015.10.007

[bib209] Siqueira-Silva T, De Lima LAG, Chaves-Silveira J, Amado TF, Naipauer J, Riul P, Martinez PA. 2021. Ecological and biogeographic processes drive the proteome evolution of snake venom. Global Ecol Biogeogr. 30:1978–89.

[bib210] Slagboom J, Kaal C, Arrahman A, Vonk FJ, Somsen GW, Calvete JJ, Wüster W, Kool J. 2022. Analytical strategies in venomics. Microchem J. 175:107187.

[bib211] Smiley-Walters SA, Farrell TM, Gibbs HL. 2019. High levels of functional divergence in toxicity towards prey among the venoms of individual pigmy rattlesnakes. Biol Lett. 15:20180876. 30958133 10.1098/rsbl.2018.0876 PMC6405466

[bib212] Smith CF, Mackessy SP. 2016. The effects of hybridization on divergent venom phenotypes: characterization of venom from *Crotalus scutulatus scutulatus* × *Crotalus oreganus helleri* hybrids. Toxicon. 120:110–23. 27496060 10.1016/j.toxicon.2016.08.001

[bib213] Smith CF, Nikolakis ZL, Ivey K, Perry BW, Schield DR, Balchan NR, Parker J, Hansen KC, Saviola AJ, Castoe TA et al. 2023a. Snakes on a plain: biotic and abiotic factors determine venom compositional variation in a wide-ranging generalist rattlesnake. BMC Biol. 21:136. 37280596 10.1186/s12915-023-01626-x PMC10246093

[bib214] Smith CF, Nikolakis ZL, Perry BW, Schield DR, Meik JM, Saviola AJ, Castoe TA, Parker J, Mackessy SP. 2023b. The best of both worlds? Rattlesnake hybrid zones generate complex combinations of divergent venom phenotypes that retain high toxicity. Biochimie. 213:176–89. 37451532 10.1016/j.biochi.2023.07.008

[bib215] Smith SM . 1977. Coral-snake pattern recognition and stimulus generalisation by naive great kiskadees (Aves: Tyrannidae). Nature. 265:535–6.

[bib216] Strickland JL, Smith CF, Mason AJ, Schield DR, Borja M, Castañeda-Gaytán G, Spencer CL, Smith LL, Trápaga A, Bouzid NM et al. 2018. Evidence for divergent patterns of local selection driving venom variation in Mojave rattlesnakes (*Crotalus scutulatus*). Sci Rep. 8:17622. 30514908 10.1038/s41598-018-35810-9 PMC6279745

[bib217] Sunagar K, Jackson T, Undheim E, Syed A, Antunes A, Fry B. 2013. Three-fingered RAVERs: rapid accumulation of variations in exposed residues of snake venom toxins. Toxins. 5:2172–208. 24253238 10.3390/toxins5112172 PMC3847720

[bib218] Sunagar K, Khochare S, Laxme RRS, Attarde S, Dam P, Suranse V, Khaire A, Martin G, Captain A. 2021. A wolf in another wolf’s clothing: post-genomic regulation dictates venom profiles of medically-important cryptic kraits in India. Toxins, 13:69. 33477742 10.3390/toxins13010069 PMC7832344

[bib219] Sunagar K, Moran Y. 2015. The rise and fall of an evolutionary innovation: contrasting strategies of venom evolution in ancient and young animals. PLoS Genet. 11:e1005596. 26492532 10.1371/journal.pgen.1005596 PMC4619613

[bib220] Sunagar K, Morgenstern D, Reitzel AM, Moran Y. 2016. Ecological venomics: how genomics, transcriptomics and proteomics can shed new light on the ecology and evolution of venom. J Proteomics. 135:62–72. 26385003 10.1016/j.jprot.2015.09.015

[bib221] Sunagar K, Undheim EAB, Scheib H, Gren ECK, Cochran C, Person CE, Koludarov I, Kelln W, Hayes WK, King GF et al. 2014. Intraspecific venom variation in the medically significant Southern Pacific rattlesnake (*Crotalus oreganus helleri*): biodiscovery, clinical and evolutionary implications. J Proteomics. 99:68–83. 24463169 10.1016/j.jprot.2014.01.013

[bib222] Sweet SS . 2016. Chasing flamingos: toxicofera and the misinterpretation of venom in varanid lizards. In: Proceedings of the 2015 Interdisciplinary World Conference on Monitor Lizards. Bangkok, Thailand: Institute for Research and Development, Suan Sunandha Rajabhat University.

[bib223] Tadokoro T, Modahl CM, Maenaka K, Aoki-Shioi N. 2020. Cysteine-rich secretory proteins (CRISPs) from venomous snakes: an overview of the functional diversity in a large and underappreciated superfamily. Toxins. 12:175. 32178374 10.3390/toxins12030175 PMC7150914

[bib224] Tan CH, Tan KY, Tan NH. 2016. Revisiting *Notechis scutatus* venom: on shotgun proteomics and neutralization by the “bivalent” sea snake antivenom. J Proteomics. 144:33–8. 27282922 10.1016/j.jprot.2016.06.004

[bib225] Tan CH, Tan KY, Yap MKK, Tan NH. 2017. Venomics of *Tropidolaemus wagleri*, the sexually dimorphic temple pit viper: unveiling a deeply conserved atypical toxin arsenal. Sci Rep. 7:43237. 28240232 10.1038/srep43237 PMC5327433

[bib226] Tan KY, Tan CH, Fung SY, Tan NH. 2015. Venomics, lethality and neutralization of *Naja kaouthia* (monocled cobra) venoms from three different geographical regions of Southeast Asia. J Proteomics. 120:105–25. 25748141 10.1016/j.jprot.2015.02.012

[bib227] Tasima LJ, Lima EOVD, Hatakeyama DM, Vidueiros JP, Stuginski DR, Grego KF, 2024. Seasonality in *Crotalus durissus* venom. Toxicon. 244:107748. 38710309 10.1016/j.toxicon.2024.107748

[bib228] Tasoulis T, Isbister G. 2017. A review and database of snake venom proteomes. Toxins. 9:290. 28927001 10.3390/toxins9090290 PMC5618223

[bib229] Tasoulis T, Lee MSY, Ziajko M, Dunstan N, Sumner J, Isbister GK. 2020. Activity of two key toxin groups in Australian elapid venoms show a strong correlation to phylogeny but not to diet. BMC Evol Biol. 20:9. 31931699 10.1186/s12862-020-1578-x PMC6958663

[bib230] Teixeira CDFP, Fernandes CM, Zuliani JP, Zamuner SF. 2005. Inflammatory effects of snake venom metalloproteinases. Mem Inst Oswaldo Cruz. 100:181–4. 15962120 10.1590/s0074-02762005000900031

[bib231] Tian H, Liu M, Li J, Xu R, Long C, Li H, Mwangi J, Lu Q, Lai R, Shen C. 2020. Snake C-type lectins potentially contribute to the prey immobilization in *Protobothrops mucrosquamatus* and *Trimeresurus stejnegeri* venoms. Toxins. 12:105. 32041262 10.3390/toxins12020105 PMC7076790

[bib232] Tioyama EC, Bayona-Serrano JD, Portes-Junior JA, Nachtigall PG, De Souza VC, Beraldo-Neto E, Grazziotin FG, Junqueira-de-Azevedo ILM, Moura-da-Silva AM, Freitas-de-Sousa LA. 2023. The venom composition of the snake tribe Philodryadini: ‘omic’ techniques reveal intergeneric variability among South American racers. Toxins. 15:415. 10.3390/toxins15070415 37505684 PMC10467154

[bib233] Trummal K, Tõnismägi K, Paalme V, Järvekülg L, Siigur J, Siigur E. 2011. Molecular diversity of snake venom nerve growth factors. Toxicon. 58:363–8. 21801740 10.1016/j.toxicon.2011.07.005

[bib234] Ullah A, Masood R . 2020. The sequence and three-dimensional structure characterization of snake venom phospholipases B. Front Mol Biosci. 7(175):1–11. 32850964 10.3389/fmolb.2020.00175 PMC7419708

[bib235] Utkin YN . 2019. Last decade update for three-finger toxins: newly emerging structures and biological activities. WJBC. 10:17–27. 30622682 10.4331/wjbc.v10.i1.17 PMC6314878

[bib236] Vaiyapuri S, Wagstaff SC, Watson KA, Harrison RA, Gibbins JM, Hutchinson EG. 2010. Purification and functional characterisation of rhiminopeptidase A, a novel aminopeptidase from the venom of *Bitis gabonica rhinoceros*. PLoS Negl Trop Dis. 4:e796. 20706583 10.1371/journal.pntd.0000796 PMC2919393

[bib237] Van Thiel J, Alonso LL, Slagboom J, Dunstan N, Wouters RM, Modahl CM, Vonk FJ, Jackson TNW, Kool J. 2023. Highly evolvable: investigating interspecific and intraspecific venom variation in taipans (*Oxyuranus* spp.) and brown snakes (*Pseudonaja* spp.). Toxins. 15:74. 36668892 10.3390/toxins15010074 PMC9864820

[bib238] Van Thiel J, Khan MA, Wouters RM, Harris RJ, Casewell NR, Fry BG, Kini RM, Mackessy SP, Vonk FJ, Wüster W et al. 2022. Convergent evolution of toxin resistance in animals. Biol Rev. 97:1823–43. 35580905 10.1111/brv.12865 PMC9543476

[bib239] Vink S, Jin AH, Poth KJ, Head GA, Alewood PF. 2012. Natriuretic peptide drug leads from snake venom. Toxicon. 59:434–45. 21147145 10.1016/j.toxicon.2010.12.001

[bib240] Vogel C-W, Fritzinger DC. 2010. Cobra venom factor: structure, function, and humanization for therapeutic complement depletion. Toxicon. 56:1198–222. 20417224 10.1016/j.toxicon.2010.04.007

[bib241] Voss RS . 2013. Opossums (Mammalia: Didelphidae) in the diets of Neotropical pitvipers (Serpentes: Crotalinae): evidence for alternative coevolutionary outcomes?. Toxicon. 66:1–6. 23402839 10.1016/j.toxicon.2013.01.013

[bib242] Voss RS, Jansa SA. 2012. Snake-venom resistance as a mammalian trophic adaptation: lessons from didelphid marsupials. Biol Rev. 87:822–37. 22404916 10.1111/j.1469-185X.2012.00222.x

[bib243] Walker AA, Robinson SD, Hamilton BF, Undheim EAB, King GF. 2020. Deadly proteomes: a practical guide to proteotranscriptomics of animal venoms. Proteomics. 20:1900324. 10.1002/pmic.201900324 32820606

[bib244] Wang B, Wang Q, Wang C, Wang B, Qiu L, Zou S, Zhang F, Liu G, Zhang L. 2020. A comparative analysis of the proteomes and biological activities of the venoms from two sea snakes, *Hydrophis curtus* and *Hydrophis cyanocinctus*, from Hainan, China. Toxicon. 187:35–46. 32871160 10.1016/j.toxicon.2020.08.012

[bib245] Werner RM, Vick JA. 1977. Resistance of the opossum (*Didelphis virginiana*) to envenomation by snakes of the family Crotalidae. Toxicon. 15:29–32. 841589 10.1016/0041-0101(77)90066-6

[bib246] Williams V, White J. 1992. Variation in the composition of the venom from a single specimen of *Pseudonaja textilis* (common brown snake) over one year. Toxicon. 30:202–6. 1557789 10.1016/0041-0101(92)90473-i

[bib247] Zancolli G, Baker T, Barlow A, Bradley R, Calvete J, Carter K, De Jager K, Owens J, Price J, Sanz L et al. 2016. Is hybridization a source of adaptive venom variation in rattlesnakes? A test, using a *Crotalus scutulatus* × *viridis* hybrid zone in southwestern New Mexico. Toxins. 8:188. 27322321 10.3390/toxins8060188 PMC4926154

[bib248] Zancolli G, von Reumont BM, Anderluh G, Caliskan F, Chiusano ML, Fröhlich J, Hapeshi E, Hempel B-F, Ikonomopoulou MP, Jungo F et al. 2024. Web of venom: exploration of big data resources in animal toxin research. GigaScience. 13: giae054 10.1093/gigascience/giae054 39250076 10.1093/gigascience/giae054 PMC11382406

[bib249] Zdenek CN, Chowdhury A, Haw GYH, Violette A, Fourmy R, Christ T, Vonk FJ, Fry BG. 2022. Taxon-selective venom variation in adult and neonate *Daboia russelii* (Russell’s viper), and antivenom efficacy. Toxicon. 205:11–9. 34752826 10.1016/j.toxicon.2021.11.004

[bib250] Zelanis A, Menezes MC, Kitano ES, Liberato T, Tashima AK, Pinto AFM, Sherman NE, Ho PL, Fox JW, Serrano SMT. 2016. Proteomic identification of gender molecular markers in *Bothrops jararaca* venom. J Proteomics. 139:26–37. 26941108 10.1016/j.jprot.2016.02.030

[bib251] Zhao H-Y, He N, Sun Y, Wang Y-C, Zhang H-B, Chen H-H, Zhang Y-Q, Gao J-F. 2023. Phylogeny-related variations in venomics: a test in a subset of habu snakes (*Protobothrops*). Toxins. 15:350. 37235384 10.3390/toxins15050350 PMC10223207

[bib252] Zheng Y, Wiens JJ. 2016. Combining phylogenomic and supermatrix approaches, and a time-calibrated phylogeny for squamate reptiles (lizards and snakes) based on 52 genes and 4162 species. Mol Phylogenet Evol. 94:537–47. 26475614 10.1016/j.ympev.2015.10.009

